# Beacon-Related Parameters of Bluetooth Low Energy: Development of a Semi-Automatic System to Study Their Impact on Indoor Positioning Systems

**DOI:** 10.3390/s19143087

**Published:** 2019-07-12

**Authors:** Gabriele Salvatore de Blasio, José Carlos Rodríguez-Rodríguez, Carmelo R. García, Alexis Quesada-Arencibia

**Affiliations:** Institute for Cybernetics, University of Las Palmas de Gran Canaria, Campus de Tafira, 35017 Las Palmas de Gran Canaria, Spain

**Keywords:** indoor positioning, bluetooth 5, bluetooth low energy, fingerprinting, semi-automatic data acquisition

## Abstract

Indoor positioning systems (IPS) are used to locate people or objects in environments where the global positioning system (GPS) fails. The commitment to make bluetooth low energy (BLE) technology the leader in IPS and their applications is clear: Since 2009, the Bluetooth Special Interest Group (SIG) has released several improved versions. BLE offers many advantages for IPS, e.g., their emitters or beacons are easily deployable, have low power consumption, give a high positioning accuracy and can provide advanced services to users. Fingerprinting is a popular indoor positioning algorithm that is based on the received signal strength (RSS); however, its main drawbacks are that data collection is a time-consuming and labor-intensive process and its main challenge is that positioning accuracy is affected by various factors. The purpose of this work was to develop a semi-automatic data collection support system in a BLE fingerprinting-based IPS to: (1) Streamline and shorten the data collection process, (2) carry out impact studies by protocol and channel on the static positioning accuracy related to configuration parameters of the beacons, such as transmission power (*Tx*) and the advertising interval (*A*), and their number and geometric distribution. With two types of systems-on-chip (SoCs) integrated in Bluetooth 5 beacons and in two different environments, our results showed that on average in the three BLE advertising channels, the configuration of the highest *Tx* (+4 dBm) in the beacons produced the best accuracy results. However, the lowest *Tx* (−20 dBm) did not worsen them excessively (only 11.8%). In addition, in both scenarios, when lowering the density of beacons by around 42.7%–50%, the error increase was only around 8%–9.2%.

## 1. Introduction

Indoor positioning systems (IPS) have been the subject of much research in recent years, mainly because the global positioning system (GPS) signal suffers from attenuation in many indoor environments, rendering it impossible to use for positioning [1], and because they have multiple and useful applications in real life [2].

There are many technologies used in IPS, such as Wi-Fi, bluetooth low energy (BLE), ZigBee, visible light or earth magnetic field [3], but none leads the field [4]. Among all these technologies, BLE is one of the most widely used in ubiquitous computing and in many Internet of things (IoT) applications [5,6,7,8]. 

BLE or bluetooth smart was introduced in 2009 as an extension to the Bluetooth 4.0 Core Specification (Bluetooth Classic) and was designed to support the IoT [9,10]. Subsequently, improved versions 4.1 and 4.2 were introduced in 2013 and 2014, which mainly introduced higher data range, higher packet capacity and much higher-strength secure connections, that is, the BLE signal reaches a greater distance, transmits more data per second and the connections are safer. Both BLE and its predecessor, Bluetooth Classic, operate in the 2.4 GHz Industrial, Scientific and Medical (ISM) radio band but the former uses only forty 2 megahertz-wide channels (as opposed to the seventy-nine 1 megahertz-wide channels used by the latter), divided into three primary advertising channels and 37 secondary advertising and data channels. Bluetooth Classic can handle a lot of data continuously in connections of about 100 ms; however, BLE is used when it is not necessary to exchange a lot of data continuously in connections of about 1 millisecond, which leads to a lower consumption of BLE compared to Bluetooth Classic.

The use of BLE technology applied to indoor positioning (IP) has many advantages: The beacons or emitters are portable, battery-powered, small, lightweight, have low energy consumption, give a high positioning accuracy and are easily deployable at low cost [11,12]. In addition, they can provide users with advanced services that are directly related to the positioning [13]. There are two relevant parameters in the configuration of BLE beacons—the transmission power (*Tx*), measured in decibel–milliwatts (dBm), and the advertising interval (*A*), measured in milliseconds (ms)—that determine, respectively, how far and how often a beacon can transmit. Both parameters have a direct impact on the battery life of the beacons: Higher values of *Tx* or lower values of *A* imply more battery discharge.

BLE beacons transmit data packets, which are slightly different for some standards or protocols, such as Apple’s iBeacon (iB) and Google’s Eddystone (Ed). These data packets can be sent in the advertising mode (AdvM), that is, regularly emitted to other listening devices, in the connection mode or in a one-to-one connection type and, finally, in the periodic advertisement mode, which allows a device that only receives advertising events (scanner or observer) to be synchronized with the advertisements sent continuously by a device that only sends advertising events (broadcaster) [14]. Of these three modes, perhaps the most used for positioning purposes is the first: Messages sent by the beacons in AdvM hop between a fixed sequence of three narrow channels, namely advertising channels or primary advertising channels 37, 38 and 39, to gain redundancy in order to reduce interference with other wireless technologies, especially with Wi-Fi channels 1, 6 and 11, commonly used in many environments [11,15]. Primary advertising channels are used for device discovery, connection establishment and broadcast transmissions. It is important to note that smartphones currently receive the aggregate signal of the three advertising channels, which may lead to reduced positioning accuracy [16].

The important novelties in Bluetooth 5 focus on the BLE version [14,17]. Bluetooth 5 provides up to 4× the range, 2× the speed and 8× the broadcasting message capacity, along with enhancements that increase functionality for the IoT. The types of connections and features supported are BLE 4.x, Bluetooth 5 at a bit rate of 2 megabits per second (Mbps), bluetooth coded and advertising extensions: The first is the connection specified by BLE 4.0, 4.1 and 4.2 at 1 Mbps, the second is the new high-speed connection, the third is for long range communications at low bit rates and the last is a way to advertise more data than is allowed with advertisements from previous versions of BLE (Legacy Advertisements). Bluetooth 5.1 was introduced in January 2019 [18]. The major feature is the possibility of absolute positioning in three-dimensional space through the angle of arrival (AoA) and angle of departure (AoD), which offer precision of direction in addition to the distance-only information that received signal strengths (RSS) traditionally brought [19].

There are different wireless indoor measuring principles, such as triangulation, scene analysis or fingerprinting and proximity [20]. Among these, fingerprinting is perhaps the most popular due to its simplicity: It is based on the intensity of the signal and its process basically consists of collecting the signal from the emitters and associating it with a particular position [1,20]. It consists of two phases: The calibration, training or offline phase and the positioning or online phase. In the calibration phase (CPh) or training phase (TPh), a site inspection or survey is carried out, collecting the received signal strengths (RSS) from the different transmitters or beacons at spatial points of known coordinates, called reference points (RP). Each RP is then characterized by a signal pattern or fingerprint. The set of fingerprints associated with all the RPs is stored in the RP-database (RP-DB) or radio map. In the positioning phase (PPh), a user at a test point (TP) of unknown coordinates measures the signals coming from the different emitters and compares these signals with those obtained in the CPh through some matching algorithm, ultimately obtaining his position. The set of fingerprints associated with all the TPs is stored in the TP-database (TP-DB). The most common fingerprinting matching algorithms can be classified as [4]: (a) Deterministic, such as the nearest neighbor (NN) and its variants k-nearest neighbor (kNN) and weighted k-nearest neighbor (WkNN) [21,22], (b) probabilistic [23] and (c) machine learning and compressive sensing-based [24].

Fingerprinting also has some drawbacks, the main one being that CPh is a time-consuming and labor-intensive process [25]. Regardless of the technology used, there are several approaches to data collection in the fingerprinting CPh, which can be classified as:

1. Those that consist of a complete CPh, e.g., the traditional manual survey, where a user collects the signal at discrete and evenly distributed survey points [26].
2. Those that attempt by some means to reduce the time and effort of the CPh, e.g., path surveys, in which, by means of Gaussian processes, a dedicated user attempts to construct a signal map from a sparse set of fingerprints collected while walking through a space [27], or interpolation-based methods, such as inverse distance weighted (IDW), radial basis function (RBF) or Kriging, which build a more populated RP-database from a few RPs [28,29].
3. Those that are CPh-free, which are subdivided into various categories, such as those that use online RSS measurements only or those that, in some manner, merge CPh and PPh using people, e.g., explicit or implicit crowdsourcing, in which users are involved in data collection, differing between them in the user incentive and degree of participation [1,25].

The main challenge in fingerprinting-based IPS is that accuracy is affected by several factors, such as signal-related and environment-related factors. With respect to the signal-related factors, the following can be mentioned: Reflection, refraction, path loss, large fluctuations, multipath fading, non-line-of-sight (NLOS) conditions, bearing in mind that many of these factors also depend on the type of material present in the environment [30,31]. With respect to environment-related factors, the main factors are changes in hardware/furniture, presence of people or ambient humidity conditions [1,32,33,34].

An automatic system would have many benefits in various fingerprinting data collection approaches: e.g., quick and easy construction of datasets, in-depth studies on the position and density of beacons in a particular environment, studies on beacon parameters, quick maintenance of the RP-DB upon hardware/furniture changes, studies on weather conditions and their impact on positioning, etc.

The main contributions of this work are:

1. We present a new semi-automatic system that, in addition to facilitating the studies mentioned below, also shortens the duration of data collection.
2. We provide studies by protocol and channel on parameters related to the configuration of beacons that can affect the static positioning accuracy, such as *Tx*, *A*, and also provide studies on the number of beacons and their geometric distribution in the deployment environment.
3. We provide studies on the impact on accuracy of decreasing the testbed grid density and increasing the number of orientations in the CPh.
4. We provide a performance study of three Bluetooth 5 systems-on-chip (SoCs): nRF52832 and nRF52810 integrated, respectively, in two different brands of beacons and nRF52840 integrated in a BLE 5 receptor, also providing studies on the impact of the horizontal/vertical receiver orientation on accuracy.

This paper is organized into six Sections: Section 2 reviews related works, Section 3 and Section 4 will describe material and methods and the experimental setup. Section 5 shows the results of the tests carried out and in Section 6 we discuss the results and future lines of work.

## 2. Related Works

In this section we will analyze the works related to the study presented in this article. First, we will give an overview of the most used technologies in IP, focusing later on those that use radio frequency (RF) signals and, with more detail, BLE.

The technologies used in IP are diverse [3]: Optical, such as those that use visible light communication (VLC), sound-based, such as those that use ultrasound, those that use RF, such as the wireless local area network (WLAN) or bluetooth, or those that rely on naturally occurring signals, such as those that use the Earth’s natural magnetic field.

With respect to VLC applied to IP, we can mention the work of Pathak et al. [35] where, among other issues, they analyzed the application of this technology to localization. A pioneer work on ultrasound technology applied to IP is that of Ward et al. [36] where the localization process was conducted through an array of microphones, tags carried by users and trilateration. An emerging technology applied to IP is magnetic positioning: In this field, we may mention the pioneer work of Haverinen et al. [37], which proposed an approach to dynamic localization using magnetic distortion inside buildings.

RF-based technologies applied to IP may be the field with the most works. One of the first was that of Bahl et al. [26] using a combination of WLAN and fingerprinting, addressing, among other important issues, the variation of RSS depending on the user’s orientation. This work was followed by many other important ones, such as those of Feng et al. [38] in which the authors used the theory of compressive sensing, or that of Kushki et al. [31] whose system, based on kernels, presented improvements compared to others that are widely used. Regarding other works based on RF technologies, we can mention that of Fang et al. [39], performed on a realistic ZigBee sensor network, or that of Ni et al. [40] which pioneered radio frequency identification (RFID)-based systems. Regarding ultra wideband (UWB), which is an emerging technology in IP, Alarifi et al. [41] provided a detailed comparative analysis of UWB positioning technologies.

Focusing primarily but not exclusively on IPS using BLE technology, we divided these related works into three sections: (1) Generic works, (2) works whose subject matter is related to automatic or semi-automatic data collection systems and (3) works that study the configuration and placement of beacons.

### 2.1. Generic Works 

Regarding works in which the viability of BLE technology in IP is analyzed, we can mention that of Kajioka et al. [42] and Faragher et al. [11]. In the first, in a 10.5 m × 15.6 m room they installed 22 BLE beacons reaching a correct position estimation rate of 96.6% for a portable device. In the second, the authors distributed 19 beacons in a 600 m^2^ testbed to position a consumer device: They demonstrated the high susceptibility of BLE to fast fading, investigated key parameters in a BLE positioning system, such as the beacon density, *Tx* or *A*, achieving for two different densities errors of around 2.6 m and 4.8 m 96% of the time, which were better than the results obtained with a Wi-Fi network. Neburka et al. [43] studied the performance of BLE for indoor localization purposes using RSS in an ideal and a real environment. To explore this performance, a simulation model based on ray-tracing was proposed, proving that BLE technology is a promising technique for indoor positioning.

Zou et al. [44] proposed BlueDetect as an accurate, fast response and energy-efficient scheme for indoor–outdoor detection and seamless location-based services running on the mobile device based on iBeacon technology: Their scheme provides precise indoor–outdoor detection results to turn on/off on-board sensors smartly, improve their performance and reduce the power consumption of mobile devices simultaneously; seamless location-based services, such as positioning and navigation services, can be realized. Zhuang et al. [16] proposed an algorithm based on the integration of a channel-separate polynomial regression model, channel-separate fingerprinting, extended Kalman filtering and outlier detection for indoor localization using BLE beacons. It provided accuracies of less than 2.56 m 90% of the time with dense deployment of BLE beacons and less than 3.99 m 90% of the time with sparse deployment of beacons. Powar et al. [45] showed that different RSS behavior in typical environments of the three BLE advertising channels almost always has a significant effect on the aggregate signal, as well as significant implications for positioning. Their analysis indicates that a single channel signal is highly preferable to a composite signal for positioning purposes; they show that a fingerprinting scheme that uses a signal map for each advertising channel achieves significantly increased positioning accuracy (up to 3 m).

The aim of the work of Tosi et al. [46] is to review the main methodologies adopted to investigate BLE performance: They provided an analysis of throughput, maximum number of connectable sensors, power consumption, latency and maximum reachable range, with the aim of identifying the current limits of BLE technology. Contreras et al. [47] evaluated the viability of BLE for indoor positioning scenarios: They showed experimentally that, with proper configuration of the BLE devices, great performance can be obtained in terms of discovery time and energy consumption.

There are several works on types of effort reduction in the fingerprinting approach. Among them, we can cite those based on WLAN technology, such as that of Chai et al. [48], which for a probabilistic location determination approach, tried to solve the problem of the large number of training samples required for calibration, reducing both the sampling time and the number of locations sampled in constructing a radio map. Their tests show that manual effort can be reduced substantially while a high level of accuracy is still achieved. King et al. [49] carried out an interesting study on the deployment, calibration and measurement factors for position errors by systematically investigating the number of access points, the number of samples in the TP, the number of samples in the PPh, and the setup of the grid of reference points. Ficco et al. [50] described an automatic tuning approach for positioning systems with the aim of reducing the manual calibration efforts. They proposed a simulation model of the signal propagation, in order to compute the RSSs without the on-field measurements, as well as to determine the better RP configuration; the approach was tested on the most used radio frequency-based technologies. In the work of Gao et al. [27], they used what they call path surveys: An attempt to construct signal maps from a sparse set of Wi-Fi and BLE fingerprints collected while a person walks through a space. Using Gaussian processes, their results show that a path survey can provide maps of equivalent quality to a manual survey. Subedi et al. [51] used BLE technology to propose a localization technique that reduces the number of required fingerprint RPs by more than 40%, compared to a normal fingerprinting localization method and with a similar localization estimation error. Zuo et al. [52] proposed a graph optimization-based way of estimating the beacon positions and the RP-radio map without any dedicated surveying instruments. In both a dense (one beacon per 69 m^2^) and a sparse beacon situation (one beacon per 137.5 m^2^), their fingerprinting-based method, which adopts an estimated RP-radio map, gives higher mean positioning errors (2.78–4.11 m) than constructing the RP-radio map by exhaustive surveying as the authors in [16] did. Sadowski et al. [53] compared Wi-Fi, BLE, ZigBee and the long range wide area network (LoRaWAN) for use in an indoor localization system. They proved Wi-Fi to be the most accurate, followed by BLE, but BLE was also found to use the lowest amount of power.

Finally, there are the works that involve Bluetooth 5 in some way. Due to its recent introduction, we can only mention that of Karvonen et al. [54], whose main goal was to evaluate experimentally the communications range and throughput performance of BLE 5 coded version, and that of Pancham et al. [55], which identified BLE as one of the technologies that promise an acceptable response to the requirements of the healthcare environment and investigated in that context the latest improvements with Bluetooth 5, especially with regard to its range when the signal penetrates through different types of multiple partitions.

### 2.2. Automatic or Semi-Automatic Data Collection Systems

With respect to automatic or semi-automatic data collection systems, Peng et al. [56] proposed a fast and efficient location fingerprint database (DB) construction and updating method, based on a self-developed unmanned ground vehicle platform: A smartphone was installed on that platform for collecting indoor RSS fingerprints, such as bluetooth and Wi-Fi. They showed that, compared with the traditional point collection and line collection schemes, the root mean square error of the fingerprinting-based positioning results were reduced by 35.9% and 25.0% in static tests and by 30.0% and 21.3%, respectively, in dynamic tests. Nastac et al. [57] addressed the problem of automatic data collection for the purpose of IP via RSS fingerprinting: A robotic platform with basic odometer sensors was used in a building to automate the process of data acquisition. Their results proved the advantage of using a robotic platform by reducing time spent in data acquisition from 16 down to two hours for 3000 observations, and the accuracy of measurement is increased due to the automatic process based on the robotic platform compared to the manual approach. De Blasio et al. [58] studied the impact and the interplay of configuration parameters related to BLE beacons in static indoor positioning as well as the orientation effect in the CPh: To reduce the data collection process, a semi-automatic system was introduced.

### 2.3. Configuration Parameters and Number/Placements of Beacons

Bulusu et al. [59] emphasized the importance of beacon placement in localization approaches and explained the need for empirically adaptive beacon placement algorithms: In their simulations, beacon density rather than noise level has a higher impact on the performance of beacon placement algorithms. Chawathe et al. [60] addressed the problem of beacon placement: They formalized the problem combinatorially as the problem of finding a maximum-resolution sub-hypergraph. An important feature of their approach is that the shape and size of the range of each beacon is completely arbitrary and may be specified based on the observed characteristics.

Ji et al. [61] analyzed the relationship between the number of installed beacons and their positioning accuracy: In a 100 m × 100 m space and using a path loss model, they deployed between 10 and 100 virtual BLE beacons in a random and in a grid manner. The simulations in the case of random beacon topology show that with more beacons the accuracy is better, although for a smaller number of beacons the values are close, while in the case of grid topology the performance is better with a dense beacon deployment. Kriz et al. [62] designed and implemented a distributed system for acquisition of a combination of Wi-Fi and BLE fingerprints: They tested several configurations of positions of transmitters (beacons) or their density and the influence of the scanning duration on the accuracy of the localization. Their results show that it is possible to improve the median accuracy by 23% and to reduce the variance. He et al. [63] defined the beacon deployment for positioning, formulated as an integer linear programming problem, and provided several theoretic bounds on the number of required beacons for unambiguous positioning: Their analysis and experiments show that their solution requires 2–8 times fewer beacons, compared to a naïve approach.

Castillo-Cara et al. [64] identified in a simple setup the main system parameters to be taken into account for the design of BLE 4.0 beacon-based indoor localization mechanisms, and explored two parameters: Transmission power and the physical characteristics of the scenario. They introduced a novel approach based on the use of an asymmetric transmission power setting of the beacons and concluded that an asymmetric transmission power setting may prove useful on mitigating the information to be provided to the classification algorithms due to the multipath fading effect. Paterna [65] proposed and implemented a real IPS based on a reduced number of BLE beacons that improves accuracy while reducing power consumption and costs. The three main proposals are channel diversity to reduce RSS dispersion, Kalman filtering to eliminate unwanted RSS values and an improved trilateration method. The analysis of the results proves that all the proposals improve the precision of the system (0.7 m 90% of the time for static devices). Falque et al. [65] proposed a novel cost-function that optimizes both the number of beacons and their placement in a given environment: Their approach accounts for RF signal attenuation due to the environment and is independent of the localization algorithm used and the results show that 53% fewer beacons are needed.

Castillo-Cara et al. [64] identified in a simple setup the main system parameters to be taken into account for the design of BLE 4.0 beacon-based indoor localization mechanisms, and explored two parameters: Transmission power and the physical characteristics of the scenario. They introduced a novel approach based on the use of an asymmetric transmission power setting of the beacons and concluded that an asymmetric transmission power setting may prove useful on mitigating the information to be provided to the classification algorithms due to the multipath fading effect. Paterna [65] proposed and implemented a real IPS based on a reduced number of BLE beacons that improves accuracy while reducing power consumption and costs. The three main proposals are channel diversity to reduce RSS dispersion, Kalman filtering to eliminate unwanted RSS values and an improved trilateration method. The analysis of the results proves that all the proposals improve the precision of the system (0.7 m 90% of the time for static devices). Falque et al. [65] proposed a novel cost-function that optimizes both the number of beacons and their placement in a given environment: Their approach accounts for RF signal attenuation due to the environment and is independent of the localization algorithm used and the results show that 53% fewer beacons are needed.

## 3. Materials and Methods

Different tests were performed in two testbeds described in the following section. In these tests, a different number of Kontakt Pro beacons or Minew E7 beacons, based on Nordic Semiconductors SoCs nRF52832 and nRF52810, respectively, were used as Bluetooth 5 emitters. Even though both SoCs support high-speed connection at a bit rate of 2 Mbps and long-range communications, the firmware versions installed on both brands of beacons used in our tests only allow connection at a bit rate of 1 Mbps. Table 1 shows the codes for the different values of *Tx* in both brands of beacons. From now on we will refer to the value of *Tx* by its corresponding codes.

**Figure 1:**
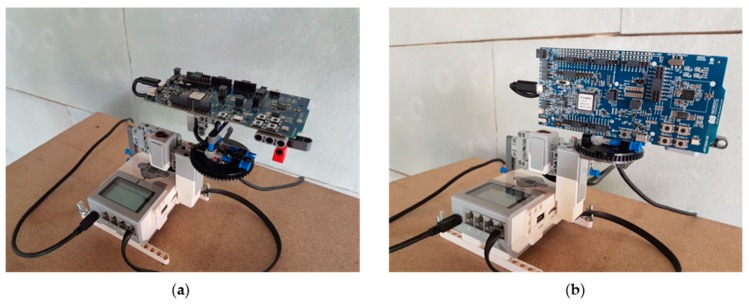
Views of the nRF52840 DK receiver coupled to the orienting device (OD): (**a**) Horizontally and (**b**) vertically.

An ASUS VivoBook X540U laptop with a Nordic Semiconductor nRF52840 BLE Development Kit (nRF52840 DK), based on SoC nRF52840, was used to collect the BLE signals. The nRF52840 DK was fixed on an orienting device (OD; see Figure 1a,b) and connected to the laptop port via a USB cable [58].

The OD allows a receiver (nRF52840 DK in this case) to be aligned automatically with a certain angle on the XY plane (or ground plane) with a resolution of 1°. The receiver can be attached horizontally or vertically to the OD (see Figure 1a,b). For the construction of the OD, parts of the Lego MindStorms EV3 Education kit were used [58,65]. The main parts were:

1. EV3 unit: Small computer that controls the motors and receives information from the sensors.
2. Servo Motor: According to the manufacturer, it offers accuracy to within 1°.
3. 8-tooth gear mounted on the motor shaft.
4. 56-tooth gear mounted on the receiver.
5. A light/color sensor: Used to calibrate the OD.

The control algorithm was written in Python running under an ev3dev (modified Debian) operating system on the EV3 unit [66]. In the CPh/PPh, the receiver is placed in a preset orientation of 0° using a light/color sensor. In the operation phase, from the previously obtained preset orientation, it responds to the specific orientation request received from the PC via USB. The motor encoder was used to estimate the position of the receiver at any time.

**Figure 2:**
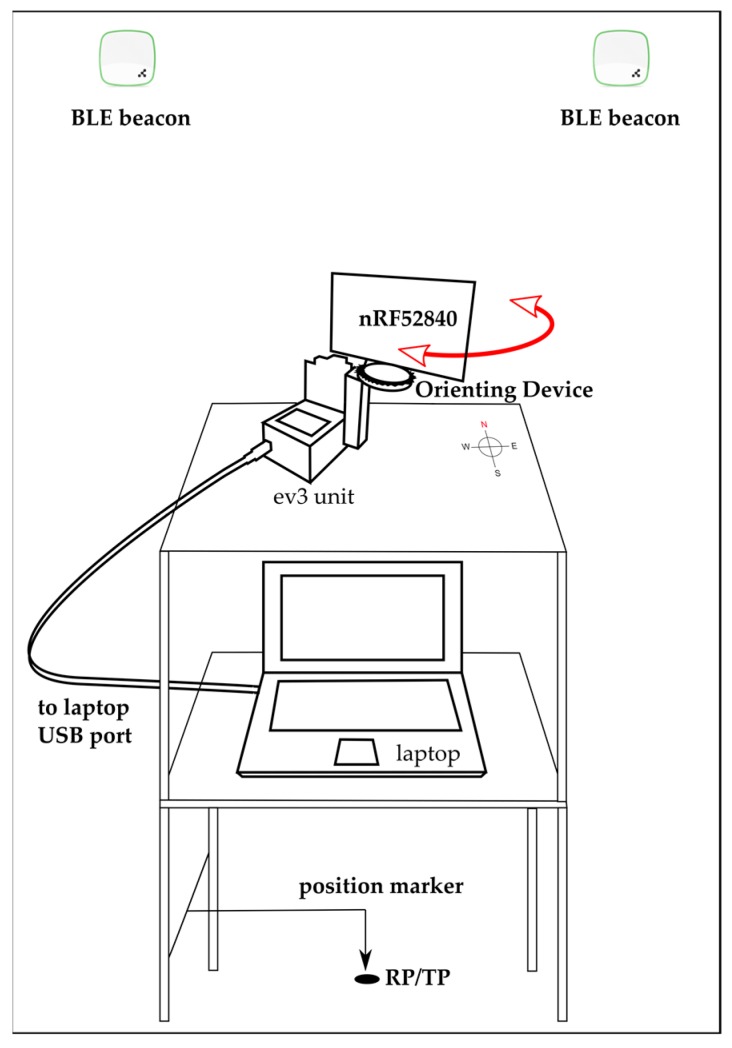
Block diagram of the acquisition platform showing the EV3 unit with the orienting device (OD), the receiver (nRF52840 DK) and the laptop computer.

Depending on the test, the nRF52840 DK was placed horizontally or vertically. The acquisition platform consists of an EV3 unit attached to a wheeled table at a height similar to that of a person holding a mobile device, the orientation of the table being fixed at all times (see Figure 2).

**Figure 3:**
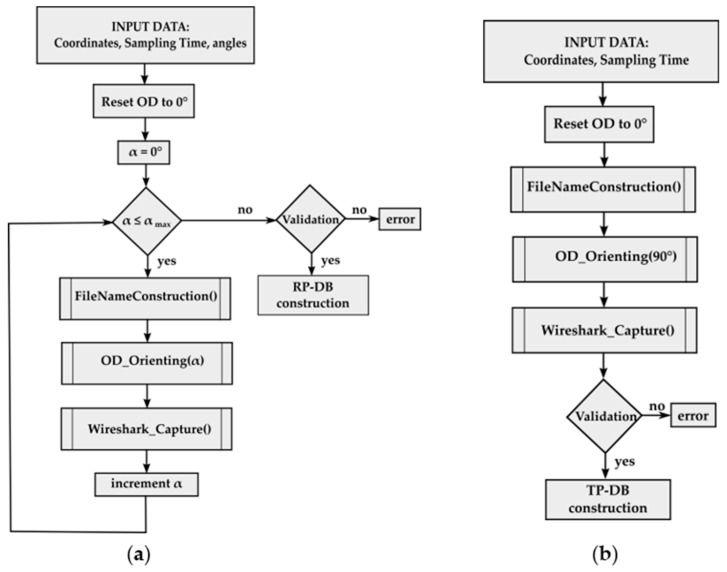
Algorithms for the data collection process: (**a**) Reference points-database (RP-DB) and (**b**) Test points-database (TP-DB).

The sniffing software employed was Nordic Semiconductor nRF52Sniffer v2.0.0-beta3 together with Wireshark 2.6.1. The data collection process was initiated through a batch file that controls the orienting device and calls the sniffing software. Figure 3a,b show the algorithm for the data collection process, depending on the database to be generated. In the case of the RP-DB, the coordinates of the particular RP, the sampling time and the angles at which the OD will place the receiver are entered as input data. In the case of the TP-DB, the coordinates of the particular RP and the sampling time are entered as input data. The OD is reset to 0° and a process begins in which the receiver is oriented by the different prefixed angles, taking the different data (mainly RSS, protocol, channel and MAC address) of each beacon through Wireshark. The data are validated at the end of the process, and if there are no capture errors, these values as well as the angles are inserted in the RP-DB. In the case of TP-DB, the process is similar but, in this case, the receiver is only situated in a specific orientation: In the tests carried out it was 90° or the north direction.

For a particular test and both in the CPh and the PPh, the same brand of beacons, laptop, receiver and software were used. WKNN was the pattern-matching algorithm used to compare fingerprints in both phases. WKNN is an improvement on the classic NN and kNN algorithms: RPs obtained in the CPh, which are closer to TPs obtained in the PPh, should have a higher weight than RPs that are far away. The estimated coordinates $\left(x_{e},y_{e}\right)$ of the TPs are calculated using the equation (1):

(1) $$\left(x_{e},y_{e}\right)=\frac{\sum_{i=1}^{k}\left(x_{i},y_{i}\right)\cdot w_{i}}{\sum_{i=1}^{k}w_{i}}\;\;\;\;\;\;w_{i}=\frac{1}{d_{i}},$$

where $\left(x_{i},y_{i}\right)$ are the coordinates of the k-RP and $w_{i}$ are the weights for each distance $d_{i}$.

**Figure 4:**
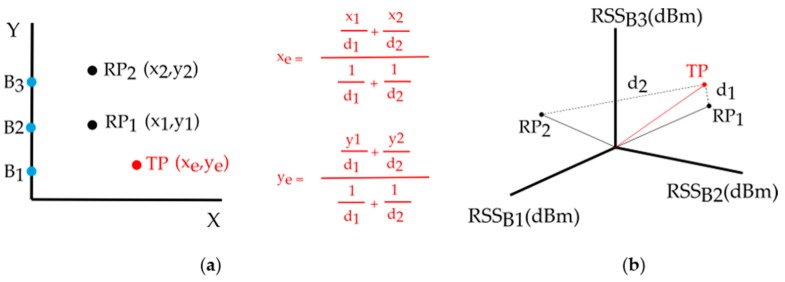
Example of application of the weighted k-nearest neighbor (WKNN) algorithm for two reference points, RP_1_ and RP_2_, one test point, TP, and three beacons, B_1_, B_2_ and B_3_: (**a**) Calculation of the estimated coordinates $\left(x_{e},y_{e}\right)$ as a weighted average of the coordinates of k-nearest RPs (*k* = 2) with the shortest distance to the TP in the signal space; (**b**) Euclidean distances of the RPs to the TP in the signal space.

Figure 4a shows a simple example of application of the WKNN algorithm for two reference points RP_1_ and RP_2_, one test point, TP, and three beacons labeled B_1_, B_2_, and B_3_. The RSS value (dBm) of each beacon is measured at each RP/TP: These values can be represented in the signal space (see Figure 4b), that is, for a particular RP or TP the RSS of all beacons is represented as a point in a multi-dimensional space. In this space, the distance $d_{i}\;\left(i=1,2\right)$ from each RP to the TP is calculated by means of a distance or similarity metric, such as the Euclidean distance of equation (2):

(2) $$d_{i}=\sqrt{{\left(RSS_{B1}^{RPi}-RSS_{B1}^{TP}\right)}^{2}+{\left(RSS_{B2}^{RPi}-RSS_{B2}^{TP}\right)}^{2}+{\left(RSS_{B3}^{RPi}-RSS_{B3}^{TP}\right)}^{2}},$$

where $RSS_{Bj}^{RPi}\;\left(j=1,2,3\right)$ is the RSS from the beacon $B_{j}$ measured at reference point $RP_{i}$ and $RSS_{Bj}^{TP}$ is the RSS from beacon $B_{j}$ measured at test point TP. In this context, the main assumption in the WKNN algorithm is that the RPs closest to the TP in the signal space (having a smaller distance, $d_{i}$) are spatially close points. Consequently those RPs will have larger weights $w_{i}$ (see Equation (1)) and a larger contribution to the final value of the estimated coordinates $\left(x_{e},y_{e}\right)$ of the TP. The distance/similarity metric employed in this paper was the Euclidean [4,67].

## 4. Experimental Setup

**Figure 5:**
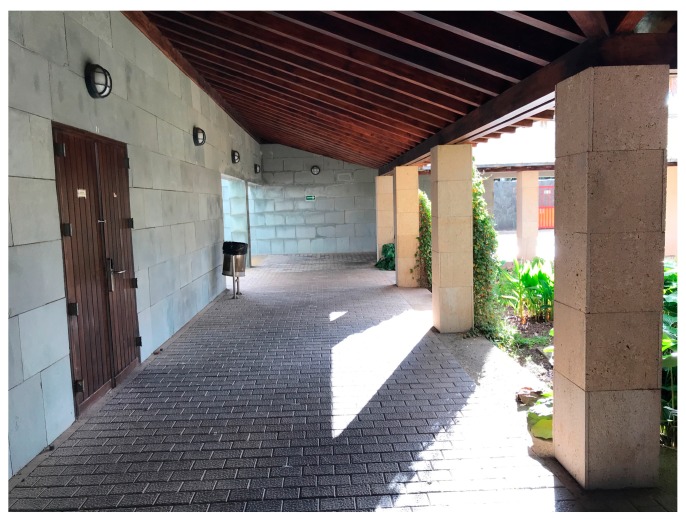
View of the first scenario where TB1 is located.

**Figure 6:**
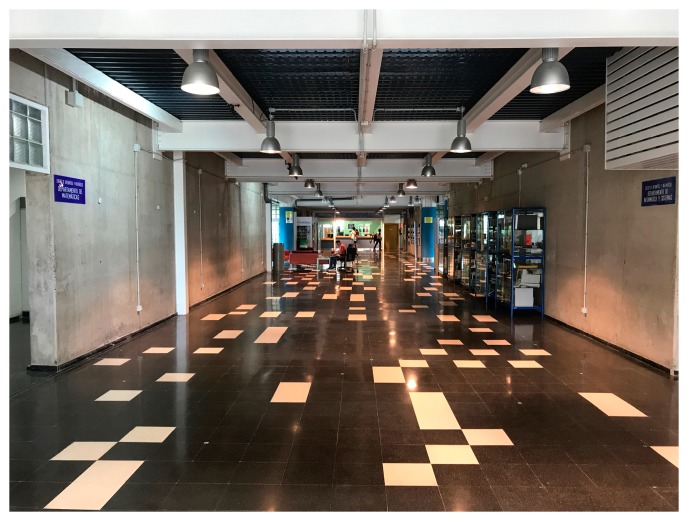
View of the second scenario where TB2 is located.

**Figure 7:**
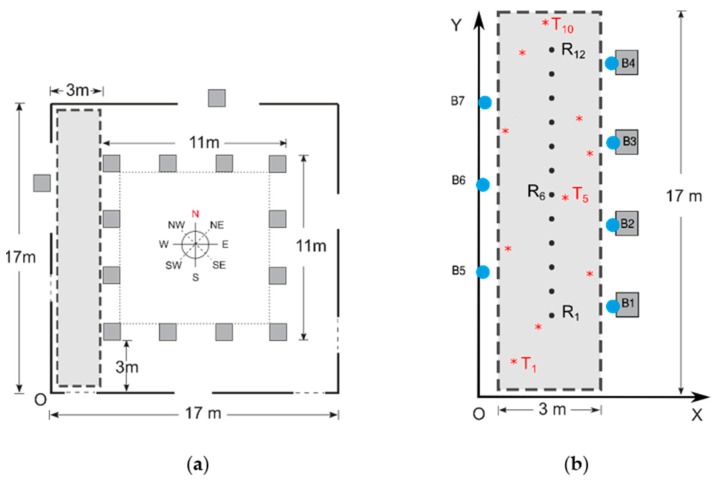
Schematic view of the first scenario: (**a**) Dimensions and orientations, (**b**) beacons, RP and TP positions.

**Figure 8:**
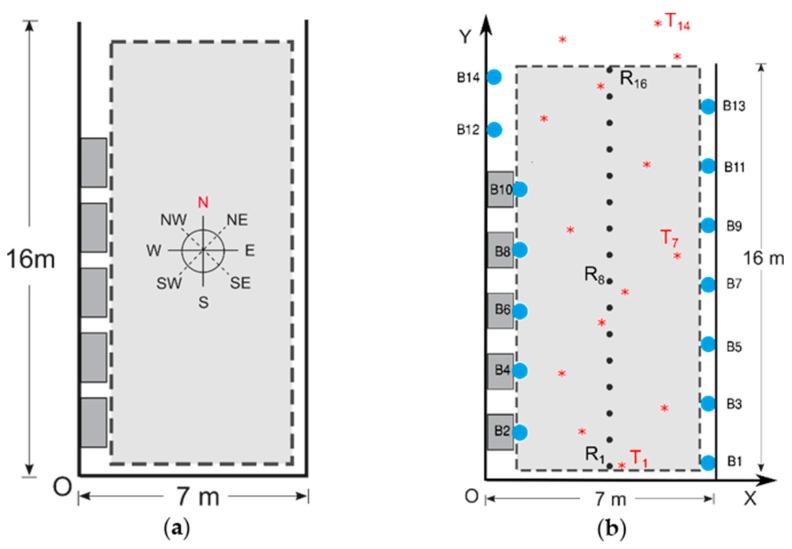
Schematic view of the second scenario: (**a**) Dimensions and orientations, (**b**) beacons, RP and TP positions.

Figure 5 and Figure 6 show the two chosen scenarios, while Figure 7 and Figure 8 show the corresponding schematic views. Following the manufacturers’ installation recommendations [68], deployed BLE 5 beacons were situated in both scenarios at a fixed height of 2.1 m and configured with the Eddystone and iBeacon protocols.

In the first scenario, part of the main corridor of our research institution, a rectangular testbed (hereinafter TB1) of 17 m × 3 m with an area of 51 m^2^ was chosen, with a grid of 12 reference points in a single line and 10 test points (see Figure 7), taking the (*x*, *y*) coordinates of all points with a laser pointer. The materials of which TB1 and its furniture are composed are mainly concrete floors and walls, wooden ceilings and some metallic trash bins. Depending on the test, four or seven beacons were installed giving densities of one beacon per 12.75 m^2^ and 7.28 m^2^ respectively. The first four (labeled B1 to B4 in Figure 7b) were placed on columns with a distance of 3.5 m between each of them, while the remaining three (labeled B5 to B7) were placed on the opposite wall with the same distance between them and at a certain displacement with respect to the first four: The objective of this configuration is that the signals from the beacons cover the largest possible surface area. All the beacons were configured with the same values of *Tx* and *A* for all tests.

In the CPh for all tests, RSS values were taken for the four cardinal directions and the four intermediate directions, i.e., east, northeast, north, northwest, west, southwest, south and southeast (E, NE, N, NW, W, SW, S and SE. See Figure 7a and Figure 8a). In some tests, both in CPh and PPh, measurements were taken without the presence of people, while in other tests a slight presence of people was included in the PPh (see Section 5.2).

The second chosen scenario was a corridor near the access to the library in the School of Computer Engineering of the University of Las Palmas de Gran Canaria. In that scenario, a rectangular testbed (hereinafter TB2) of 16 m × 7 m with an area of 112 m^2^ was chosen with a grid of 16 RPs in a single line and 14 TPs (see Figure 8), taking the (*x*, *y*) coordinates of all points with a laser pointer. The materials of which TB2 and its furniture are composed of mainly ceramic floors, concrete walls, metal and glass showcases and metal ceilings. Fourteen beacons were installed giving a density of one beacon per 8.0 m^2^. The first seven, labeled with odd numbers (B1 to B13 of Figure 8b), were placed on a wall with a distance of 2.15 m between each of them, while the remaining seven, labeled with even numbers (B2 to B14), were placed on the opposite side, some on top of the showcases (B2 to B12) and some on top of a wall (B13, B14), with the same distance between them and a certain displacement with respect to beacons on the opposite wall: The objective was the same as in TB1. All the beacons were configured with the same values of *Tx* and *A* for all tests. In this scenario, the movement of people during the tests through the testbed was continuous in both phases (see Section 5.9, Section 5.10 and Section 5.11).

Once the raw reference fingerprints had been recorded with 15 s of samples (TB1) or 30 s of samples (TB2), a smaller fingerprint database was constructed taking the mean of the maximum RSS values for each beacon, cardinal direction, protocol and channel [69]. In the PPh, a similar procedure used in the CPh was used to record at 14 TPs situated randomly in the grid: In this case, and in order to simulate a real positioning situation, only eight samples for each protocol and channel in a fixed orientation were taken (north direction). A smaller fingerprint database was constructed from the original: For each beacon, protocol and channel, the maximum RSS value was calculated in the fixed orientation. We guaranteed the coherence of the orientation in both phases using a compass attached to the wheeled table.

The ideal and theoretical CPh-duration for each test is given by equation (3):

(3) $$D=t_{s}\cdot O\cdot R,$$

where $t_{s}$ is the sampling time in each RP per orientation, O is the number of orientations in the CPh and R is the number of RPs. In the particular cases of testbeds TB1 and TB2, the values of D were, respectively, 12–24 min (6–12 RPs) and 32–64 min (8–16 RPs).

## 5. Results 

In this work, positioning accuracy and precision were expressed, respectively, by the mean error and the cumulative distribution function (CDF) obtained through the WKNN algorithm [20]. Euclidean was the distance used for the calculation of weights in the WKNN algorithm since in previous works it has been detected that the distance/similarity used is not critical [69]. As was mentioned in Section 3, the current firmware versions installed in the two brands of Bluetooth 5 beacons only support a 1 Mbps bit rate, therefore, this was the default value for all tests. Although the specific value of *A* = 100 ms consumes more battery in the beacons than other higher values, it has been used in many tests because it considerably reduces site survey time by allowing sufficient samples to be obtained in a short time interval and because increasing the CPh sampling time does not necessarily increase accuracy significantly [49,69]. Nevertheless, in some tests the *A* = 500 ms value was also employed. In order to reduce the CPh, we also tested lower radio map densities by increasing the distance between RPs: Increasing the radio map density provides better accuracies but only to a certain extent [28].

**Figure 9:**
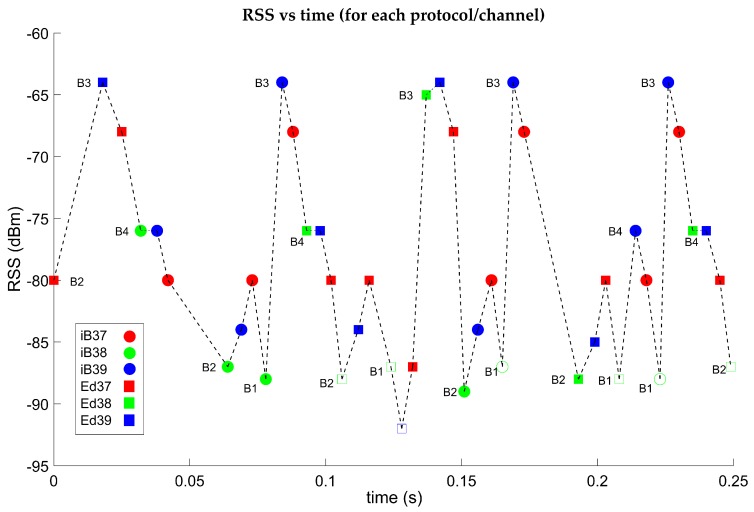
Received signal strength (RSS) values vs. time for each protocol, channel and beacon. Dots represent iBeacon and squares Eddystone protocols respectively.

We carried out some studies prior to the tests. In the first, we represented the RSS values vs. time for each protocol, channel and beacon. Figure 9 shows the case corresponding to the test of Section 5.1. In this image, several things were observed: The receiver capturing data packets coming from the beacons follows in general the sequence of channels 37, 38, 39, independently of the protocol and beacon. It was also observed that the packets that Wireshark reports as erroneous (empty circles or squares) corresponded to very low RSS values: These packets were ruled out in all tests.

In the second, we studied how the number of samples varies as the number of protocols and beacons increases. It is easy to see that the theoretical number of samples, s, per protocol (iBeacon or Eddystone) and channel is given by the relation expressed by equation (4):

(4) $$s=\frac{10^{3}\cdot t_{s}}{A}\;,$$

where $t_{s}$ is the sampling time (in seconds) and *A* is the advertising interval (in ms). The total number of samples in the three channels will be $s_{T}=3\;s$. We detected that when progressively increasing progressively the number of protocols and beacons, the value of $s_{T}$ is almost equally distributed between the two protocols, and more importantly, is smaller but very close to the theoretical value of Equation (4). Table 2 and Table 3 show the theoretical and experimental values of $s_{T}$ for a specific case.

This result will allow us in some tests to simulate a smaller number of beacons with certain geometrical distributions by removing from the RP-DB and the TP-DB the values corresponding to the beacons to eliminate. In the case of having actually done the tests with a lower number of beacons, the number of samples would be equal to or greater than the number obtained by these simulations, so we can state that in any case the results obtained correspond to an unfavorable situation with respect to the number of samples.

**Figure 10:**
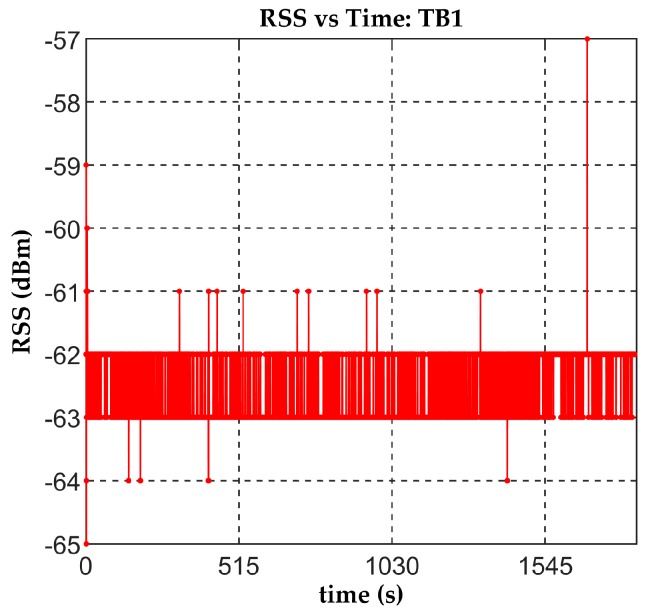
Evolution of RSS during one hour for TB1.

In the third preliminary study, we took the RSS values of seven beacons in testbed TB1 during one hour at an intermediate point on the grid. The objective of this study was to observe the temporal behavior of the BLE signal and its fast fades in order to filter out the outliers if necessary. Figure 10 shows the RSS vs. time plot: As there were no people present during this preliminary test, the signal was almost constant but there were outliers of up to 6 dBm. In some of the subsequent tests, we filtered outliers that were more than three scaled median absolute deviations.

In all the tests presented in the next subsections, the data collection at each RP was carried out in eight directions on the *XY* plane (see Figure 7a and Figure 8a): In a previous work we observed better positioning accuracy when increasing the number of directions in the CPh [58].

Tests performed in TB1 are described below in Section 5.1 to 5.8, while those performed in TB2 are described in Section 5.9 to 5.11. In each subsection, a description of the experiment with its objectives and parameters is introduced. As a summary, Table 4 shows the main configuration of each test, the scenario in which it was carried out and the objective of the test. Finally, Section 5.12 summarizes the conclusions of each test.

### 5.1. TESTS #1: TB1, nRF52840 DK Attached Horizontally to OD

The aim of this group of tests was to test the effect of *Tx* on the positioning accuracy for a specific orientation of the receiver (nRF52840 DK). Figure 1a shows the way in which the receiver was initially attached to the OD. Table 5 shows the main features of this group of tests.

Kontakt Pro beacons B1 to B4 (see Figure 7b) were configured with three different values of *Tx* and a unique value of *A*. In both phases, no people were present during the data collection. Table 6 shows the accuracy results. We will present only the best accuracy values (first three k-neighbors) although in all tests performed the best results were obtained using *k* = 1.

In general, it was observed that *Tx* code 7 produced better results for accuracy, although in some channels the differences were small; therefore, in the following tests only the two extreme *Tx* values of the beacons were used, that is, *Tx* code 1 and *Tx* code 7.

### 5.2. TESTS #2a and #2b: TB1, nRF52840 DK Attached Vertically to OD

In order to test the effect of receiver orientation, nRF52840 DK was attached vertically to the OD in this group of tests (see Figure 1b). 

Test #2a. No people were present during the test in both phases. Table 7 shows the main features of these tests. 

Table 8 shows accuracy results for Test #2a. 

Comparing values of Table 6 and Table 8 it could be clearly seen that when the receiver was attached vertically to the OD, accuracy improved, with *Tx* code 7 (+4 dBm) giving the best overall results. 

Test #2b. As an additional test to #2a, we included a slight noise in the environment with the presence of two people in the positioning phase for *Tx* code 7: The first person standing at each RP/TP just in front of the receiver (blocking the signal coming from the beacons situated behind) and the second person walking continuously around the environment. Table 9 shows a comparison of accuracy values.

It can be seen that the slight noise introduced in the PPh had an influence on the positioning accuracy but was not excessive.

All the following tests were carried out with the nRD52840 DK attached vertically to OD.

### 5.3. TEST #3: TB1, MINEW E7 Beacons

In order to compare the bit rate performance of SoC 52,832 and SoC 52810, MINEW E7 beacons B1 to B4 (see Figure 7b) were configured with unique *Tx* and *A* values. Table 10 shows the main features of this test.

Table 11 shows accuracy results for Test #3.

Observing the values in Table 11, it can be seen that with *Tx* code 7, SoC nRF52832 integrated in Kontakt Pro beacons offered better results (except for channel 38) than SoC nRF52810 integrated in MINEW E7 beacons.

The following tests were carried out only with Kontak Pro beacons.

### 5.4. TESTS #4: TB1, Increasing the Number of Beacons

The aim of these two tests was to study the effect on accuracy by increasing the number of beacons. Kontakt Pro beacons B1 to B7 (see Figure 7b) were configured with two extreme values of *Tx* and a unique value of *A*. Table 12 shows the main features of these tests.

The values in Table 8 and Table 13 show that increasing the number of beacons from four to seven had a positive impact on accuracy for both values of *Tx*, although it was more relevant in channels 38 and 39.

Table 13 shows accuracy results for Tests #4.

### 5.5. TESTS #5: TB1, Variable Number and Geometric Distribution of Beacons

The results of Section 5.4 led us to study the impact of the number of beacons and their geometric distribution on the accuracy. Using the DB constructed in Section 5.4., a variable number of beacons and geometric distribution was simulated by eliminating the RSS values of some particular beacons in the RP-DB and TP-DB. It is important to note that these tests are based on previous results for the number of samples seen at the beginning of Section 5. Table 14 shows the main features of these tests.

Table 15 and Table 16 show accuracy results for *Tx* code 1 and *Tx* code 7 respectively. 

In general, it was observed that the best accuracy was obtained as the number of beacons increased although in some cases the values were similar. 

### 5.6. TESTS #6a and #6b: TB1, Higher Advertising Interval, A.

The previous results led us to study the impact of increasing the value of *A* while keeping the sampling time constant, which entails reducing the number of samples. Table 17 shows the main features of these tests and Table 18 shows a comparison of the accuracy for two values of *A*.

It was observed that reducing the advertising interval (from *A* = 100 ms to *A* = 500 ms) did not have an excessive impact on accuracy.

As we did in a previous experiment (see Section 5.2) we included a slight noise with the presence of two people in the test field. Table 19 shows accuracy results for Tests #6.

As in the test mentioned above, introducing a slight noise did not have a great impact on accuracy.

### 5.7. TESTS #7: TB1, Lower Grid Density

The purpose of these tests was to check the effect on the accuracy of a grid with fewer reference points: In particular, odd reference points RP1, RP3, etc. were taken (see Figure 7b). Table 20 shows the main features of these tests. 

Table 21 shows accuracy results for Tests #7.

Comparing accuracy values of Table 21 it could be seen that halving grid density did not excessively worsen the accuracy for both *Tx* code 1 and *Tx* code 7.

### 5.8. TESTS #8: TB1, Seven Beacons, Removal of Outliers

As discussed in previous studies at the beginning of Section 5, we filtered outliers that were more than three scaled median absolute deviations. Table 22 shows a comparison of accuracies with and without removing outliers.

It could be observed that the introduction of an outlier filtering step had little influence on the accuracy in general.

In the next subsections, we will describe the tests performed in the second testbed (TB2).

### 5.9. TESTS #9: TB2, 14 Beacons

In order to validate the results obtained in the first testbed (TB1), we carried out a set of tests in the second testbed (TB2).

As we saw in Section 4, the area of this test field is 112 m^2^ and a density of beacons equal to one beacon per 8 m^2^ was initially used. Regarding the presence of people, two people were standing at each RP/TP just in front of the receiver (blocking the signal coming from the beacons situated behind) and, on average, five people were crossing the testbed.

Table 23 and Table 24 show the main features and accuracy results of these tests, respectively.

**Figure 11:**
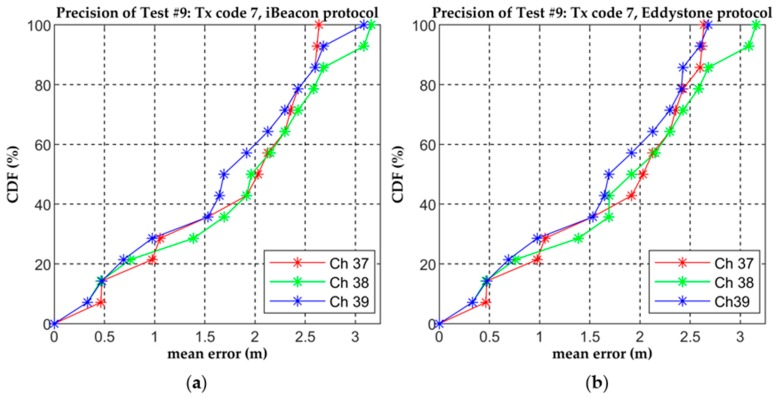
Precision of Test #9 for *Tx* code 7, three advertising channels and (**a**) iBeacon protocol and (**b**) Eddystone protocol.

From Table 24 it can be seen that the accuracy results were better for *Tx* code 7 although for some channels the differences were relatively small. Figure 11 shows the precision of this test for *Tx* code 7 represented by the value of the CDF vs. the mean error.

### 5.10. TESTS #10: TB2, Variable Number and Geometric Distribution of Beacons

Table 25 shows the main features of these tests while Table 26 and Table 27 show accuracies for *Tx* code 1 and *Tx* code 7, respectively. Regarding the presence of people, two people were standing at each RP/TP just in front of the receiver (blocking the signal coming from the beacons situated behind) and, on average, five people were crossing the testbed.

Just as in the tests of Section 5.5, it was observed that increasing the number of beacons improved accuracy, but the differences were not very large.

### 5.11. TESTS #11: TB2, Lower Grid Density

As we did in Section 5.7 for testbed TB1, we also studied the effect of a lower grid density in TB2. Table 28 shows the main features of these tests and Table 29 shows the corresponding accuracy. Regarding the presence of people, two people were standing at each RP/TP just in front of the receiver (blocking the signal coming from the beacons situated behind) and, on average, five people were crossing the testbed.

As in Section 5.7, it could be seen that halving grid density did not excessively worsen the accuracy for both *Tx* code 1 and *Tx* code 7.

### 5.12. Conclusions Related to the Tests 

Table 30 shows the main conclusions about accuracy for the different tests.

## 6. Discussion

The best accuracy results in both scenarios were obtained with power *Tx* code 7 and k = 1 (see Table 6 and Table 24). However, in both scenarios the results with power *Tx* code 1 were not far from these values: On average in the three channels the error only worsened by about 11.8%. This result was important because lower power means considerable battery savings, which translates into savings in the maintenance cost of a possible massive deployment of beacons. Consequently, in these scenarios and from an energy point of view, a conservative strategy could be chosen in relation to *Tx*.

The vertical or horizontal orientation of the receiving device (nRF52840 DK) had an impact on accuracy, with the best results being obtained with vertical orientation (see Table 8). On average in the three channels the improvement in accuracy was around 14.4%. Therefore, in the possible tests that we will carry out in the future with this device, the vertical orientation will be used.

Introducing a slight noise during the positioning phase did not significantly affect accuracy (see Table 9). On average in the three channels accuracy worsened when introducing noise by around 2.2%. Therefore, it was possible to collect data in scenarios with a moderate presence of people without having a significant impact on positioning.

Comparing the accuracy results of SoC nRF52832 and nRF52810, it could be seen that in general the former gave better values than the latter (see Table 11). The average improvement in accuracy in the three channels was around 2.2%. Therefore, we would opt for testing with the first SoC in future works.

Decreasing the density of beacons worsened accuracy in both scenarios. For example, in the first scenario, when the number of beacons decreased by 71% (from seven to two, that is, from a density of one beacon per 7.3 m^2^ to one beacon per 25.5 m^2^), the average accuracy in the three channels worsened by 58% (see Table 16). However, decreasing the number of beacons in TB1 by 43% (from seven to four, that is, from a density of one beacon per 7.3 m^2^ to one beacon per 12.75 m^2^), the average accuracy worsened in the three channels by only 8%. In the second, for example, when the number of beacons decreases by 50% (from 14 to seven, that is, from a density of one beacon per 8.0 m^2^ to one beacon per 16.0 m^2^), the average accuracy in the three channels worsened by only 9.2% (see Table 27). Again, in scenarios where it was necessary to maintain low energy consumption and low deployment and maintenance costs, it was possible to use a low density of beacons without excessively altering accuracy. It was observed that in both scenarios certain geometric distributions of beacons offer better results than others. New in-depth studies are needed on the influence of the geometric distribution of beacons on positioning.

Increasing the value of the advertising interval, *A*, did not have a significant impact on static positioning accuracy, whether there was a slight presence of noise or not (see Table 18 and Table 19). Multiplying the value of *A* by 5 (from *A* = 100 to *A* = 500) led to an average accuracy reduction in the three channels of only 6.1%. Again, this result tells us that in scenarios where low energy consumption and low maintenance costs are required, it is possible to increase the value of *A* without excessively altering the accuracy.

Accuracy did not worsen significantly in both scenarios by increasing the distance between reference points, i.e., lowering the resolution of the grid (an RP line in this case). For example, a 50% reduction in the number of RPs (from 12 RPs to six in the first scenario and from 16 to eight in the second) only worsened accuracy on average in the three channels by around 8% and 11.1% respectively (see Table 21 and Table 29). This result directly affected the CPh-DB construction, which is a fingerprinting drawback, and therefore in future tests will serve as a basis for initially establishing a less populated grid of RPs.

Removal of outliers in the reference points-database did not have a significant impact on improving accuracy (see Table 22).

The calibration phase in fingerprinting is a long and tedious process: The studies presented in this paper aim to facilitate and reduce the duration of data collection and to find the optimal values of parameters related to BLE beacons, such as *Tx*, *A*, number, geometric distribution, etc., which favor energy saving, achieve good static positioning accuracy and, therefore, offer precise advanced services. 

Following the results obtained in previous tests, we can conclude that best accuracy results were obtained using beacons with SoC nRF52832, receptor nRF52840 with vertical orientation and *A* = 100 ms. Using the above parameters, Table 31 shows the influence of other studied parameters on the positioning accuracy (average of the three channels) for TB2.

From these results and from an energy point of view, we can conclude that it is possible to perform a conservative deployment, i.e., with a low density of beacons with low *Tx* values, obtaining an accuracy not far from less conservative deployments (with higher densities and *Tx* values). For example, in row 4 we observed an accuracy of 2.1 m (very close to the best of 1.8 m) setting a lower *Tx* (–20 dBm) and a lower density of beacons (one per 16 m^2^).

The semi-automatic system presented allowed us to perform many tests without excessive time cost. Table 32 shows a comparison of the duration of tests performed using the proposed semi-automatic system and a manual data collection system. For the calculation of the duration of each test under ideal conditions and using the proposed semi-automatic system, Equation (3) was used, and in the case of a manual data collection, a delay of 30 s in the changes between orientations was also estimated using the same equation.

From Table 32 the benefits of the proposed semi-automatic system can be clearly seen: The duration of the tests was reduced by between 50% and 66%. These results signify a clear contribution of the presented work, since it contributes to mitigating one of the great handicaps of fingerprinting: The time that is dedicated to data collection in the CPh and that in many cases makes its use unfeasible in real environments. Moreover, these times could be shortened introducing some improvements. As stated in the introductory section, an automatic data capture system would have many benefits in various fingerprinting data collection approaches. The semi-automatic system presented is the first stage of a fully automatic platform that we are currently developing.

In addition, and with respect to other related works, the work presented here deals with the variation of RSS by increasing the number of user orientations (8) leading to an improvement in positioning accuracy.

From Table 32 and Table 33 it was also observed that a 50% reduction in the number of RPs did not excessively worsen the accuracy and produced a 50% reduction in the duration of these tests. Although it is not a simple task to compare different IPS since, in general, each system may have a different parameterseter range and a different way of computing accuracy/precision, etc., Table 33 compared the precision values obtained in other works with that obtained in our proposal. As could be seen, we achieved an accuracy of 2.6 m 90% of the time with a much lower density of RPs (0.12 RP/m^2^).

Another notable development was our study of the performance of two different BLE 5 SoCs: nRF52832 and nRF52810. These SoCs, with the right firmware, will in the future support all the features of BLE 5 mentioned in Section 1, and therefore this work could serve as a basis for other studies. With respect to positioning accuracy, our results show that the first performed slightly better than the second: On average, in the three advertising channels, the accuracy was 1.3 m vs. 1.4 m, respectively. However, in a possible situation of a massive deployment, it would be necessary to take into account the cost of each beacon that these SoCs integrate—currently 32€ and 11€, respectively.

With respect to the nRF52840 receiver, although we are aware of its use in two different works [54,55], this receiver is used for purposes other than indoor positioning. In addition, the study carried out with this receiver regarding the influence of its orientation on the accuracy of the positioning is novel; the best results were obtained with the vertical orientation.

## Figures and Tables

**Table 1:** Transmitter power (*Tx*) codes in both brands of beacons.

Tx code	1	2	3	4	5	6	7
dBm	−20	−16	−12	−8	−4	0	+4

**Table 2:** Theoretical values $s_{T}$ for 15 s of sampling time and 1000 ms of advertising interval.

$t_{s}$	A	s	$s_{T}$
15	1000	15	45

**Table 3:** Experimental value of $s_{T}$ for each of the seven beacons.

Beacon	B1	B2	B3	B4	B5	B6	B7
$s_{T}$	42	42	45	33	37	42	42

**Table 4:** Principal configuration of the tests carried out and their main objectives.

Testbed	Test #: Main Setup	Objective
TB1	#1: Receiver attached horizontally to OD (SoC nRF52832).	Effect of *Tx* on the positioning accuracy for a specific receiver orientation.
	#2a: Receiver attached vertically to OD without presence of people (SoC nRF52832).	Idem
	#2b: Idem #2a with presence of people (SoC nRF52832).	Idem
	#3: Receiver attached vertically to OD (SoC nRF52810).	Idem
	#4: Receiver attached vertically to OD with SoC nRF52832.	Effect of number of beacons on the positioning accuracy.
	#5: Idem #4.	Effect of number of beacons and geometric distribution on the positioning accuracy.
	#6a: Idem #4 without presence of people.	Effect of increasing the value of *A* on the positioning accuracy.
	#7: Idem #4.	Effect of a lower grid density on the positioning accuracy.
	#8: Idem #4	Effect of removal of outliers on the positioning accuracy.
TB2	#9: Idem #4.	Validate the positioning accuracy obtained in Test #4 for a beacon density in a different testbed.
	#10: Idem #4.	Effect of number of beacons and geometric distribution on the positioning accuracy in a different testbed.
	#11: Idem #4.	Effect of a lower grid density on the positioning accuracy in a different testbed.

**Table 5:** Main features of Tests #1.

Feature	Value
No. of RP/TP	12/10
No. of beacons/density	4/1 beacon per 12.75 m^2^
*Tx* code	1, 5, 7
*A*	100 ms
Sampling time per point (CPh/PPh)	15 s (8 samples PP)
Presence of people in CPh and/or PPh	No

**Table 6:** Accuracy (in m) for Tests #1. For each *Tx* code, the first and second data columns refer to the iBeacon and Eddystone protocol respectively. The three advertising channels are referred to as Ch 37, etc., and the number of neighbors in the WKNN algorithm, as *k*.

	*Tx* code = 1		*Tx* code = 5		*Tx* code = 7	
**k**	**Ch 37**					
**1**	1.4	1.4	1.5	1.4	1.5	1.5
**2**	1.6	1.6	1.5	1.6	1.6	1.6
**3**	1.7	1.7	1.7	1.8	1.7	1.7
	**Ch 38**					
**1**	1.9	2.0	1.8	1.6	1.7	1.7
**2**	1.8	1.9	1.7	1.8	1.6	1.6
**3**	1.9	1.9	1.9	1.9	1.8	1.8
	**Ch 39**					
**1**	2.0	1.9	2.1	2.1	1.5	1.4
**2**	2.1	2.0	1.9	2.2	1.7	1.7
**3**	1.9	1.9	1.8	2.0	1.9	1.9

**Table 7:** Main features of Tests #2a and #2b.

Feature	Value
No. of RP/TP	12/10
No. of beacons/density	4/1 beacon per 12.75 m^2^
*Tx* code	1, 7
*A*	100 ms
Sampling time per point (CPh/PPh)	15 s (8 samples PPh)
Presence of people in CPh and/or PPh	No (#2a)—Yes (#2b, PPh)

**Table 8:** Accuracy (in m) for Test #2a. For each *Tx* code, the first and second data columns refer to the iBeacon and Eddystone protocol respectively. The three advertising channels are referred to as Ch 37, etc., and the number of neighbors in the WKNN algorithm, as *k*.

	*Tx* code = 1		*Tx* code = 7	
**k**	**Ch 37**			
**1**	1.4	1.4	1.2	1.2
**2**	1.5	1.4	1.3	1.3
**3**	1.5	1.5	1.3	1.3
	**Ch 38**			
**1**	1.6	1.6	1.5	1.5
**2**	1.5	1.5	1.6	1.6
**3**	1.7	1.6	1.6	1.6
	**Ch 39**			
**1**	1.2	1.3	1.3	1.3
**2**	1.5	1.6	1.4	1.4
**3**	1.5	1.5	1.6	1.4

**Table 9:** Comparison of accuracies (in m) for Tests #2a and #2b. For each option, the first and second data columns refer to the iBeacon and Eddystone protocol respectively. The three advertising channels are referred to as Ch 37, etc., and the number of neighbors in the WKNN algorithm, as *k*.

	*Tx* code = 7 (no people)		*Tx* code = 7 (people)	
**k**	**Ch 37**			
**1**	1.2	1.2	1.4	1.4
**2**	1.3	1.3	1.3	1.4
**3**	1.3	1.3	1.4	1.5
	**Ch 38**			
**1**	1.5	1.5	1.5	1.7
**2**	1.6	1.6	1.6	1.8
**3**	1.6	1.6	1.7	1.8
	**Ch 39**			
**1**	1.3	1.3	1.2	1.3
**2**	1.4	1.4	1.3	1.3
**3**	1.6	1.4	1.3	1.4

**Table 10:** Main features of Tests #3.

Feature	Value
No. of RP/TP	12/10
No. of beacons/density	4/1 beacon per 12.75 m^2^
*Tx* code	7
*A*	100 ms
Sampling time per point (CPh/PPh)	15 s (8 samples PPh)
Presence of people in CPh and/or PPh	No

**Table 11:** Accuracies (in m) for Test #3. For each option, the first and second data columns refer to the iBeacon and Eddystone protocol respectively. The three advertising channels are referred to as Ch 37, etc., and the number of neighbors in the WKNN algorithm, as *k*.

	*Tx* code = 7 (Kontakt)		*Tx* code = 7 (MINEW)	
**k**	**Ch 37**			
**1**	1.2	1.2	1.5	1.5
**2**	1.3	1.3	1.6	1.6
**3**	1.3	1.3	1.7	1.7
	**Ch 38**			
**1**	1.5	1.5	1.3	1.3
**2**	1.6	1.6	1.3	1.3
**3**	1.6	1.6	1.5	1.5
	**Ch 39**			
**1**	1.3	1.3	1.3	1.4
**2**	1.4	1.4	1.4	1.4
**3**	1.6	1.4	1.7	1.8

**Table 12:** Main features of Tests #4.

Feature	Value
No. of RP/TP	12/10
No. of beacons/density	7/1 beacon per 7.3 m^2^
*Tx* code	1, 7
*A*	100 ms
Sampling time per point (CPh/PPh)	15 s (8 samples PPh)
Presence of people in CPh and/or PPh	No

**Table 13:** Accuracies (in m) for Tests #4. For each *Tx* code, the first and second data columns refer to the iBeacon and Eddystone protocol respectively. The three advertising channels are referred to as Ch 37, etc., and the number of neighbors in the WKNN algorithm, as *k*.

	*Tx* code = 1		*Tx* code = 7	
**k**	**Ch 37**			
**1**	1.5	1.5	1.1	1.2
**2**	1.6	1.6	1.3	1.3
**3**	1.6	1.7	1.4	1.4
	**Ch 38**			
**1**	1.4	1.4	1.3	1.4
**2**	1.3	1.4	1.4	1.3
**3**	1.5	1.5	1.4	1.4
	**Ch 39**			
**1**	1.2	1.2	1.1	1.2
**2**	1.4	1.3	1.3	1.3
**3**	1.4	1.4	1.3	1.3

**Table 14:** Main features of Tests #5.

Feature	Value
No. of RP/TP	12/10
No. of beacons/density	2–7/1 beacon per 25.5 m^2^–7.3 m^2^
*Tx* code	1, 7
*A*	100 ms
Sampling time per point (CPh/PPh)	15 s (8 samples PPh)
Presence of people in CPh and/or PPh	No

**Table 15:** Accuracies (in m) for Test #5, *Tx* code 1. For each beacon combination, the first and second data columns refer to the iBeacon and Eddystone protocol respectively. The first row shows the total number of beacons and the second shows the labels of the beacons used (see Figure 7b). The three advertising channels are referred to as Ch 37, etc., and the number of neighbors in the WKNN algorithm, as *k*.

	2 beacons		3 beacons		4 beacons		4 beacons		5 beacons		5 beacons		5 beacons		6 beacons		7 beacons	
	1,4		1,4,6		1,2,3,4		1,4,5,7		1,3,5,6,7		2,4,5,6,7		1,2,3,4,6		1,2,3,4,5,7		1,2,3,4,5,6,7	
**k**	**Ch 37**																	
**1**	2.0	2.0	1.6	1.6	1.4	1.4	1.5	1.5	1.5	1.5	1.7	1.6	1.4	1.5	1.4	1.4	1.5	1.5
**2**	2.0	1.8	1.6	1.6	1.5	1.4	1.5	1.5	1.6	1.6	1.6	1.6	1.5	1.5	1.4	1.4	1.6	1.6
**3**	1.9	1.9	1.7	1.6	1.5	1.5	1.5	1.5	1.7	1.8	1.6	1.6	1.6	1.6	1.5	1.5	1.6	1.7
	**Ch 38**																	
**1**	2.3	2.2	1.3	1.4	1.6	1.6	1.9	1.9	2.5	2.6	1.3	1.3	1.4	1.4	1.6	1.6	1.4	1.4
**2**	2.1	2.1	1.6	1.6	1.5	1.5	1.9	1.9	2.4	2.4	1.3	1.3	1.3	1.4	1.7	1.8	1.3	1.4
**3**	2.3	2.2	2.0	2.0	1.7	1.6	2.0	2.0	2.1	2.3	1.8	1.8	1.6	1.5	1.7	1.7	1.5	1.5
	**Ch 39**																	
**1**	2.1	2.3	1.4	1.4	1.2	1.3	1.6	1.4	2.5	2.4	1.3	1.2	1.2	1.2	1.4	1.4	1.2	1.2
**2**	2.1	2.0	1.4	1.4	1.5	1.6	1.4	1.4	2.0	1.9	1.3	1.3	1.4	1.4	1.4	1.3	1.4	1.3
**3**	2.1	2.1	1.5	1.5	1.5	1.5	1.5	1.5	1.9	1.8	1.4	1.3	1.4	1.4	1.5	1.5	1.4	1.4

**Table 16:** Accuracies (in m) for Test #5, *Tx* code 7. For each beacon combination, the first and second data columns refer to the iBeacon and Eddystone protocol respectively. The first row shows the total number of beacons and the second shows the labels of the beacons used (see Figure 7b). The three advertising channels are referred to as Ch 37, etc., and the number of neighbors in the WKNN algorithm, as *k*.

	2 beacons		3 beacons		4 beacons		4 beacons		5 beacons		5 beacons		5 beacons		6 beacons		7 beacons	
	1,4		1,4,6		1,2,3,4		1,4,5,7		1,3,5,6,7		2,4,5,6,7		1,2,3,4,6		1,2,3,4,5,7		1,2,3,4, 5,6,7	
**k**	**Ch 37**																	
**1**	1.7	1.7	1.2	1.2	1.2	1.2	1.7	1.7	1.4	1.3	1.3	1.3	1.1	1.1	1.2	1.2	1.1	1.2
**2**	1.7	1.7	1.4	1.3	1.3	1.3	1.7	1.6	1.5	1.5	1.3	1.3	1.3	1.3	1.4	1.4	1.3	1.3
**3**	1.6	1.7	1.4	1.4	1.3	1.3	1.7	1.9	1.6	1.6	1.4	1.4	1.4	1.4	1.3	1.4	1.4	1.4
**k**	**Ch 38**																	
**1**	2.2	2.1	2.0	1.4	1.5	1.5	1.8	1.6	1.6	1.6	1.3	1.3	1.3	1.4	1.5	1.7	1.3	1.4
**2**	2.1	2.1	1.7	1.6	1.6	1.6	1.8	1.9	2.1	2.0	1.3	1.4	1.3	1.3	1.6	1.6	1.4	1.3
**3**	2.1	2.2	1.7	1.7	1.6	1.6	1.8	2.0	1.9	1.9	1.5	1.4	1.4	1.4	1.6	1.6	1.4	1.4
**k**	**Ch 39**																	
**1**	1.9	1.9	1.5	1.3	1.3	1.3	1.9	1.8	2.4	2.4	1.2	1.2	1.1	1.2	1.6	1.6	1.1	1.2
**2**	2.0	2.0	1.5	1.5	1.4	1.4	1.8	1.8	2.4	2.4	1.3	1.3	1.3	1.3	1.7	1.7	1.3	1.3
**3**	2.0	2.0	1.5	1.5	1.6	1.4	1.8	1.8	2.2	2.2	1.3	1.5	1.3	1.4	1.6	1.7	1.3	1.3

**Table 17:** Main features of Tests #6.

Feature	Value
No. of RP/TP	12/10
No. of beacons/density	7/1 beacon per 7.3 m^2^
*Tx* code	7
*A*	500 ms
Sampling time per point (CPh/PPh)	15 s (8 samples PPh)
Presence of people in CPh and/or PPh	No (#6a)—Yes (#6b, PPh)

**Table 18:** Comparison of accuracies (in m) for Test #6a. For each value of *A*, the first and second data columns refer to the iBeacon and Eddystone protocol respectively. The three advertising channels are referred to as Ch 37, etc., and the number of neighbors in the WKNN algorithm, as *k*.

	*A* = 100		*A* = 500	
**k**	**Ch 37**			
**1**	1.1	1.2	1.2	1.2
**2**	1.3	1.3	1.3	1.3
**3**	1.4	1.4	1.4	1.4
	**Ch 38**			
**1**	1.3	1.4	1.3	1.4
**2**	1.4	1.3	1.3	1.3
**3**	1.4	1.4	1.4	1.4
	**Ch 39**			
**1**	1.1	1.2	1.2	1.2
**2**	1.3	1.3	1.3	1.3
**3**	1.3	1.3	1.3	1.3

**Table 19:** Comparison of accuracies (in m) for Tests #6a and #6b. For each option, the first and second data columns refer to the iBeacon and Eddystone protocol respectively. The three advertising channels are symbolized as Ch 37, etc. and the number of neighbors in the WKNN algorithm as *k*.

	*Tx* code = 7 (no people)		*Tx* code = 7 (people)	
**k**	**iB37**	**Ed37**	**iB37**	**Ed37**
**1**	1.2	1.2	1.1	1.1
**2**	1.3	1.3	1.3	1.3
**3**	1.4	1.4	1.4	1.3
	**iB38**	**Ed38**	**iB38**	**Ed38**
**1**	1.3	1.4	1.3	1.3
**2**	1.3	1.3	1.5	1.4
**3**	1.4	1.4	1.5	1.6
	**iB39**	**Ed39**	**iB39**	**Ed39**
**1**	1.2	1.2	1.3	1.2
**2**	1.3	1.3	1.3	1.3
**3**	1.3	1.3	1.4	1.4

**Table 20:** Main features of Tests #7.

Feature	Value
No. of RP/TP	6/10
No. of beacons/density	7/1 beacon per 7.3 m^2^
*Tx* code	1, 7
*A*	100 ms
Sampling time per point (CPh/PPh)	15 s (8 samples PPh)
Presence of people in CPh and/or PPh	No

**Table 21:** Accuracy (in m) for Tests #7. For each number of RP, the first and second data columns refer to the iBeacon and Eddystone protocol respectively. The three advertising channels are referred to as Ch 37, etc., and the number of neighbors in the WKNN algorithm, as *k*.

Tx code = 1					Tx code = 7			
	*RP* = 12		*RP* = 6		*RP* = 12		*RP* = 6	
**k**	**Ch 37**							
**1**	1.5	1.5	1.5	1.5	1.1	1.2	1.3	1.3
**2**	1.6	1.6	1.8	1.8	1.3	1.3	1.6	1.6
**3**	1.6	1.7	2.1	2.1	1.4	1.4	1.8	1.8
	**Ch 38**							
**1**	1.4	1.4	1.3	1.3	1.3	1.4	1.3	1.3
**2**	1.5	1.4	1.8	1.9	1.4	1.3	1.8	1.6
**3**	1.5	1.5	2.0	2.1	1.4	1.4	1.8	1.8
	**Ch 39**							
**1**	1.2	1.2	1.3	1.3	1.1	1.2	1.3	1.3
**2**	1.4	1.3	1.6	1.5	1.3	1.3	1.6	1.6
**3**	1.4	1.4	2.0	2.0	1.3	1.3	1.8	1.9

**Table 22:** Comparison of accuracies (in m) for Tests #4 and #8. For each option, the first and second data columns refer to the iBeacon and Eddystone protocol respectively. The three advertising channels are referred to as Ch 37, etc., and the number of neighbors in the WKNN algorithm, as *k*.

	*Tx* code = 1		*Tx* code = 1 (outl. rem.)	
**k**	**Ch 37**			
**1**	1.5	1.5	1.4	1.4
**2**	1.6	1.6	1.5	1.5
**3**	1.6	1.7	1.5	1.5
	**Ch 38**			
**1**	1.4	1.4	1.3	1.3
**2**	1.3	1.4	1.3	1.3
**3**	1.5	1.5	1.4	1.5
	**Ch 39**			
**1**	1.2	1.2	1.1	1.1
**2**	1.4	1.3	1.3	1.3
**3**	1.4	1.4	1.4	1.4

**Table 23:** Main features of Tests #9.

Feature	Value
No. of RP/TP	16/14
No. of beacons/density	14/1 beacon per 8 m^2^
*Tx* code	1, 7
*A*	100 ms
Sampling time per point (CPh/PPh)	30 s (8 samples PPh)
Presence of people in CPh and/or PPh	Yes.

**Table 24:** Accuracies (in m) for Tests #9. For each value of *A*, the first and second data columns refer to the iBeacon and Eddystone protocol respectively. The three advertising channels are referred to as Ch 37, etc., and the number of neighbors in the WKNN algorithm, as *k*.

	*Tx* code = 1		*Tx* code = 7	
**k**	**Ch 37**			
**1**	2.0	2.0	1.8	1.8
**2**	2.0	1.9	1.8	1.7
**3**	2.0	2.0	1.9	1.9
	**Ch 38**			
**1**	2.2	2.3	1.9	1.9
**2**	2.1	2.1	1.9	2.0
**3**	2.0	2.1	1.9	2.0
	**Ch 39**			
**1**	1.9	1.9	1.7	1.7
**2**	1.9	1.8	1.7	1.7
**3**	2.1	2.0	1.9	2.0

**Table 25:** Main features of Tests #10.

Feature	Value
No. of RP/TP	16/14
No. of beacons/density	7–14/1 beacon per 16 m^2^–8 m^2^
*Tx* code	1, 7
*A*	100 ms
Sampling time per point (CPh/PPh)	30 s (8 samples PPh)
Presence of people in CPh and/or PPh	Yes

**Table 26:** Accuracies (in m) for Tests #10, *Tx* code 1. For each number of beacons, the first and second data columns refer to the iBeacon and Eddystone protocol respectively. The first row shows the total number of beacons and the second shows the labels of the beacons used (see Figure 8b). The three advertising channels are referred to as Ch 37, etc., and the number of neighbors in the WKNN algorithm, as *k*.

	7 beacons		7 beacons		8 beacons		11 beacons		11 beacons		14 beacons	
	1,3,5,7,9,11,13		2,4,6,8,10,12,14		1,5,9,13 2,6,10,14		1,3,5,7,9,11,13 2,6,10,14		1,5,9,13 2,4,6,8,10,12,14		1,3,5,7,9,11,13 2,4,6,8,10,12,14	
**k**	**Ch 37**											
**1**	2.1	2.1	2.0	2.1	2.4	2.2	2.3	2.0	2.1	2.0	2.0	2.0
**2**	2.1	2.1	2.0	2.1	2.2	2.2	2.1	2.1	2.0	2.0	2.0	1.9
**3**	2.1	2.0	2.0	2.1	2.1	2.1	2.1	2.1	2.0	2.0	2.0	2.0
	**Ch 38**											
**1**	2.2	3.2	2.4	2.4	2.2	2.3	2.1	2.3	2.3	2.4	2.2	2.3
**2**	2.6	3.0	2.2	2.3	2.0	2.0	1.9	2.0	2.1	2.1	2.1	2.1
**3**	2.6	3.1	2.2	2.2	2.1	2.1	2.0	2.2	2.2	2.1	2.0	2.1
	**Ch 39**											
**1**	2.0	1.9	3.0	2.9	2.1	2.2	2.0	1.9	2.0	2.3	1.9	1.9
**2**	1.8	1.8	2.7	2.5	2.1	2.1	1.9	1.8	2.0	2.0	1.9	1.8
**3**	2.0	1.9	2.5	2.4	2.2	2.2	2.0	2.0	2.1	2.2	2.1	2.0

**Table 27:** Accuracies (in m) for Tests #10, *Tx* code 7. For each number of beacons, the first and second data columns refer to the iBeacon and Eddystone protocol respectively. The first row shows the total number of beacons and the second shows the labels of the beacons used (see Figure 8b). The three advertising channels are referred to as Ch 37, etc., and the number of neighbors in the WKNN algorithm, as *k*.

	7 beacons		7 beacons		8 beacons		11 beacons		11 beacons		14 beacons	
	1,3,5,7,9,11,13		2,4,6,8,10,12,14		1,5,9,13 2,6,10,14		1,3,5,7,9,11,13 2,6,10,14		1,5,9,13 2,4,6,8,10,12,14		1,3,5,7,9,11,13 2,4,6,8,10,12,14	
**k**	**Ch 37**											
**1**	2.1	2.0	2.1	2.1	2.0	2.0	1.9	1.9	1.8	1.8	1.8	1.8
**2**	2.2	2.0	1.9	2.4	1.9	2.0	1.9	1.8	1.9	1.8	1.8	1.7
**3**	2.2	1.9	2.2	2.2	2.0	2.0	2.0	2.0	2.0	2.0	1.9	1.9
	**Ch 38**											
**1**	2.9	2.9	1.9	2.0	2.3	2.3	2.0	2.0	2.0	2.0	1.9	1.9
**2**	2.6	2.6	2.0	2.0	2.1	2.1	1.9	1.9	2.0	2.1	1.9	2.0
**3**	2.6	2.5	2.0	2.0	2.1	2.1	1.9	1.9	2.0	2.0	1.9	2.0
	**Ch 39**											
**1**	1.9	1.9	1.9	1.9	1.8	1.8	1.7	1.7	1.8	1.7	1.7	1.7
**2**	2.0	1.9	1.8	1.9	1.8	1.7	1.7	1.7	1.8	1.7	1.7	1.7
**3**	2.0	2.1	2.1	2.2	1.8	1.9	1.9	1.9	1.8	1.9	1.9	2.0

**Table 28:** Main features of Tests #11.

Feature	Value
No. of RP/TP	8/14
No. of beacons/density	14/1 beacon per 8 m^2^
*Tx* code	1, 7
*A*	100 ms
Sampling time per point (CPh/PPh)	30 s (8 samples PPh)
Presence of people in CPh and/or PPh	Yes

**Table 29:** Accuracy (in m) for Tests #11. For each number of RP, the first and second data columns refer to the iBeacon and Eddystone protocol respectively. The three advertising channels are referred to as Ch 37, etc., and the number of neighbors in the WKNN algorithm, as *k*.

*Tx* code = 1					*Tx* code = 7			
	*RP* = 16		*RP* = 8		*RP* = 16		*RP* = 8	
**k**	**Ch 37**							
**1**	2.0	2.0	2.1	2.1	1.8	1.8	2.0	2.0
**2**	2.0	1.9	2.2	2.0	1.8	1.7	2.1	2.1
**3**	2.0	2.0	2.4	2.4	1.9	1.9	2.4	2.4
	**Ch 38**							
**1**	2.2	2.3	2.1	2.2	1.9	1.9	2.1	2.2
**2**	2.1	2.1	2.2	2.6	1.9	2.0	2.1	2.2
**3**	2.0	2.1	2.7	2.7	1.9	2.0	2.7	2.7
	**Ch 39**							
**1**	1.9	1.9	2.2	2.0	1.7	1.7	1.9	1.9
**2**	1.9	1.8	2.2	2.3	1.7	1.7	1.9	1.9
**3**	2.1	2.0	2.4	2.4	1.9	2.0	2.4	2.4

**Table 30:** Main conclusions about positioning accuracy for the different tests.

Testbed	Test #: Main Setup	Main Conclusions about Accuracy
TB1	#1: Receiver attached horizontally to OD (SoC nRF52832).	Of the two orientations in which the receiver had been arranged, the vertical was the one that offered the best results. In addition, the presence of people had not worsened the results significantly.
	#2a: Receiver attached vertically to OD without presence of people (SoC nRF52832).	
	#2b: Idem #2a with presence of people (SoC nRF52832).	
	#3: Receiver attached vertically to OD (SoC nRF52810).	The performance of SoC nRF52832 was in general the best.
	#4: Receiver attached vertically to OD with SoC nRF52832.	
	#5: Idem #4.	It was possible to reduce the number of beacons, increase the value of *A* and lower the density of the grid while keeping errors to a limit.
	#6a: Idem #4 without presence of people.	
	#7: Idem #4.	
	#8: Idem #4	Removal of outliers did not have a significant impact.
TB2	#9: Idem #4.	It was confirmed in another scenario that it was possible to reduce the number of beacons and lower the density of the grid while keeping errors to a limit.
	#10: Idem #4.	
	#11: Idem #4.	

**Table 31:** Influence of *Tx* and beacon density on positioning accuracy with beacons integrating SoC nRF52832, with a receptor nRF52840 in a vertical orientation and *A* = 100 ms.

*Tx* (dBm)	Beacon Density	Accuracy (m)
+4	1 per 8 m^2^	1.8
−20	1 per 8 m^2^	2.1
+4	1 per 16 m^2^	2.0
−20	1 per 16 m^2^	2.1
+4	1 per 12.3 m^2^	1.8
−20	1 per 12.3 m^2^	2.1

**Table 32:** Test duration comparison for a semi-automatic and a manual data collection system.

Test #	Sampling Time (s)	Number of RPs	Test Duration without Semi-Automatic System (min)	Test Duration with Semi-Automatic System (min)
#1 - #6	15	12	72	24
#7	15	6	36	12
#9 - #10	30	16	128	64
#11	30	8	64	32

**Table 33:** Precision comparison between different works including our proposal.

Work	Grid Density (RPs/m^2^)	Precision (%–m)
Kajioka [42]	0.34	96.6–0.8
Powar [45]	0.43	90–13.2
Subedi [51]	3.0	90–3.0
Castillo [64]	0.26	92.5–3.0
Presented work	0.12	90–2.6

## Abbreviations

A	Advertising Interval
AdvM	Advertising Mode
AoA	Angle of Arrival
AoD	Angle of Departure
B1	Beacon 1
BLE	Bluetooth Low Energy
CDF	Cumulative Distribution Function
CPh	Calibration Phase
DB	Database
dBm	Decibel-milliwatts
E	East
Ed	Eddystone
GPS	Global Positioning System
iB	iBeacon
IDW	Inverse Distance Weighted
IoT	Internet of Things
IP	Indoor Positioning
IPS	Indoor Positioning System
ISM	Industrial, Scientific and Medical
kNN	k-Nearest Neighbor
LoRaWAN	Long Range Wide Area Network
Mbps	Megabits per second
ms	Milliseconds
N	North
NE	Northeast
NLOS	Non-Line-Of-Sight
NN	Nearest Neighbor
nRF52840 DK	Nordic Semiconductor nRF52840 BLE Development Kit
NW	Northwest
OD	Orienting Device
PPh	Positioning Phase
RBF	Radial Basis Function
RF	Radio Frequency
RFID	Radio Frequency Identification
RP	Reference Point
RP-DB	Reference Points-Database
RSS	Received Signal Strength
S	South
SE	Southeast
SIG	Special Interest Group
SoC	System-on-Chip
SW	Southwest
TB1	Testbed 1
TB2	Testbed 2
TP	Test Point
TP-DB	Test Points-Database
TPh	Training Phase
Tx	Transmission Power
UWB	Ultra Wide Band
VLC	Visible Light Communication
W	West
WkNN	Weighted k-Nearest Neighbor
WLAN	Wireless Local Area Network

## References

[1] He S., Chan S. (2016). Wi-Fi Fingerprint-Based Indoor Positioning: Recent Advances and Comparisons. IEEE Commun. Surv. Tut..

[2] Torres-Sospedra J., Montoliu R., Trilles S., Belmonte O., Huerta J. (2015). Comprehensive analysis of distance and similarity measures for Wi-Fi fingerprinting indoor positioning systems. Expert Syst. Appl..

[3] Brena R., Garcia-Vazquez J., Galvan-Tejada C., Munoz-Rodriguez D., Vargas-Rosales C., Fangmeyer J. (2017). Evolution of Indoor Positioning Technologies: A Survey. J. Sensors.

[4] Davidson P., Piche R. (2017). A Survey of Selected Indoor Positioning Methods for Smartphones. IEEE Commun. Surv. Tut..

[5] Palattella M., Dohler M., Grieco A., Rizzo G., Torsner J., Engel T., Ladid L. (2016). Internet of Things in the 5G Era: Enablers, Architecture, and Business Models. IEEE J. Sel. Area. Comm..

[6] Gomez C., Oller J., Paradells J. (2012). Overview and Evaluation of Bluetooth Low Energy: An Emerging Low-Power Wireless Technology. Sensors.

[7] Hervas R., Fontecha J., Ausin D., Castanedo F., Lopez-de-Ipina D., Bravo J. (2013). Mobile Monitoring and Reasoning Methods to Prevent Cardiovascular Diseases. Sensors.

[8] Espinilla M., Martinez L., Medina J., Nugent C. (2018). The Experience of Developing the UJAml Smart Lab. IEEE Access.

[9] Bluetooth SIG Proprietary Bluetooth 4.0 Core Specification. https://www.bluetooth.org/docman/handlers/downloaddoc.ashx?doc_id=456433.

[10] Townsend K., Cufí C., Davidson R. (2014). Getting Started with Bluetooth Low Energy: Tools and Techniques for Low-Power Networking.

[11] Faragher R., Harle R. (2015). Location Fingerprinting With Bluetooth Low Energy Beacons. IEEE J. Sel. Area. Comm..

[12] Siekkinen M., Hiienkari M., Nurminen J., Nieminen J. How Low Energy is Bluetooth Low Energy? Comparative Measurements with ZigBee/802.15.4. https://www.eecs.umich.edu/courses/eecs589/papers/06215496.pdf.

[13] De Blasio G., Quesada-Arencibia A., Garcia C., Molina-Gil J., Caballero-Gil C. (2017). Study on an Indoor Positioning System for Harsh Environments Based on Wi-Fi and Bluetooth Low Energy. Sensors.

[14] Bluetooth SIG Proprietary Bluetooth 5 Core Specification. https://www.bluetooth.org/docman/handlers/DownloadDoc.ashx?doc_id=421043.

[15] Bui H. Bluetooth Smart and Nordic’s Softdevices-Part 1 GAP Advertising. https://devzone.nordicsemi.com/b/blog/posts/bluetooth-smart-and-the-nordics-softdevices-part-1.

[16] Zhuang Y., Yang J., Li Y., Qi L., El-Sheimy N. (2016). Smartphone-Based Indoor Localization with Bluetooth Low Energy Beacons. Sensors.

[17] Collotta M., Pau G., Talty T., Tonguz O. (2018). Bluetooth 5: A Concrete Step Forward toward the IoT. IEEE Commun. Mag..

[18] Bluetooth SIG Proprietary Core Specifications | Bluetooth Technology Website. https://www.bluetooth.org/docman/handlers/downloaddoc.ashx?doc_id=457080.

[19] Leonard J. Bluetooth 5.1 Puts Bluetooth In Its Place. https://blog.nordicsemi.com/getconnected/bluetooth-5.1-puts-bluetooth-in-its-place.

[20] Liu H., Darabi H., Banerjee P., Liu J. (2007). Survey of wireless indoor positioning techniques and systems. IEEE Trans. Syst. Man Cybern. Part C (Appl Rev.).

[21] Bailey T., Jain A.K. (1978). Note on Distance-Weighted k-Nearest Nearest Rules. IEEE T. Syst. Man. Cyb..

[22] Dudani S.A. (1976). Distance-Weighted k-Nearest-Neighbor Rule. IEEE T. Syst. Man. Cyb..

[23] Youssef M., Agrawala A., Association U. The Horus WLAN location determination system. Proceedings of the Third International Conference on Mobile Systems, Applications, and Services (MobiSys 2005).

[24] Brunato M., Battiti R. (2005). Statistical learning theory for location fingerprinting in wireless LANs. Comput. Netw..

[25] Hossain A., Soh W. (2015). A survey of calibration-free indoor positioning systems. Comput. Commun..

[26] Bahl P., Padmanabhan V.N. RADAR: An In-building RF-Based User Location and Tracking System. Proceedings of the IEEE 9th Annual Joint Conference of the IEEE Computer and Communications Societies.

[27] Gao C., Harle R. Easing the Survey Burden: Quantitative Assessment of Low-Cost Signal Surveys for Indoor Positioning. In Proceeding of the Seventh International Conference on Indoor Positioning and Indoor Navigation (IPIN 2016).

[28] Conesa J., Pérez-Navarro A., Torres-Sospedra J., Montoliu R. (2019). Geographical and Fingerprinting Data to Create Systems for Indoor Positioning and Indoor/Outdoor Navigation. Challenges, Experiences and Technology Roadmap.

[29] Li B., Wang Y., Lee H., Dempster A., Rizos C. (2005). Method for yielding a database of location fingerprints in WLAN. IEE Proc.–Commun..

[30] Rappaport T.S. (2001). Wireless Communications: Principles and Practice.

[31] Buehrer R.M., Zekavat R. (2012). Handbook of Position Location. Theory, Practices and Advances.

[32] Paterna V., Auge A., Aspas J., Bullones M. (2017). A Bluetooth Low Energy Indoor Positioning System with Channel Diversity, Weighted Trilateration and Kalman Filtering. Sensors.

[33] Kaemarungsi K., Krishnamurthy P., Society I.C. Properties of indoor received signal strength for WLAN location fingerprinting. Proceedings of the First International Conference on Mobile and Ubiquitous Systems: Networking and Systems (MobiQuitous 2004).

[34] Stella M., Russo M., Begusic D. (2012). RF Localization in Indoor Environment. Radioengineering.

[35] Pathak P., Feng X., Hu P., Mohapatra P. (2015). Visible Light Communication, Networking, and Sensing: A Survey, Potential and Challenges. IEEE Commun. Surv. Tut..

[36] Ward A., Jones A., Hopper A. (1997). A new location technique for the active office. IEEE Pers. Commun..

[37] Haverinen J., Kemppainen A. (2009). Global indoor self-localization based on the ambient magnetic field. Robot. Auton. Syst..

[38] Feng C., Au W., Valaee S., Tan Z. (2012). Received-Signal-Strength-Based Indoor Positioning Using Compressive Sensing. IEEE Trans. Mob. Comput..

[39] Fang S., Wang C., Huang T., Yang C., Chen Y. (2012). An Enhanced ZigBee Indoor Positioning System With an Ensemble Approach. IEEE Commun. Lett..

[40] Ni L., Liu Y., Lau Y., Patil A. (2004). LANDMARC: Indoor location sensing using active RFID. Wirel. Netw..

[41] Alarifi A., Al-Salman A., Alsaleh M., Alnafessah A., Al-Hadhrami S., Al-Ammar M., Al-Khalifa H. (2016). Ultra Wideband Indoor Positioning Technologies: Analysis and Recent Advances. Sensors.

[42] Kajioka S., Mori T., Uchiya T., Takumi I., Matsuo H. Experiment of Indoor Position Presumption Based on RSSI of Bluetooth LE Beacon. Proceedings of the IEEE 3rd Global Conference on Consumer Electronics (GCCE 2014).

[43] Neburka J., Tlamsa Z., Benes V., Polak L., Kaller O., Bolecek L., Sebesta J., Kratochvil T., Stas J., Pleva M. Study of the Performance of RSSI based Bluetooth Smart Indoor Positioning. Proceedings of the 26th International Conference Radioelektronika (RADIOELEKTRONIKA).

[44] Zou H., Jiang H., Luo Y., Zhu J., Lu X., Xie L. (2016). BlueDetect: An iBeacon-Enabled Scheme for Accurate and Energy-Efficient Indoor-Outdoor Detection and Seamless Location-Based Service. Sensors.

[45] Powar J., Gao C., Harle R. Assessing the Impact of Multi-Channel BLE Beacons on Fingerprint-based Positioning. Proceedings of the Eigth International Conference on Indoor Positioning and Indoor Navigation (IPIN 2017).

[46] Tosi J., Taffoni F., Santacatterina M., Sannino R., Formica D. (2017). Performance Evaluation of Bluetooth Low Energy: A Systematic Review. Sensors.

[47] Contreras D., Castro M., de la Torre D. (2017). Performance evaluation of bluetooth low energy in indoor positioning systems. Trans. Emerg. Telecommun. Technol..

[48] Chai X., Yang Q. (2007). Reducing the calibration effort for probabilistic indoor location estimation. IEEE T. Mobile Comput..

[49] King T., Haenselmann T., Effelsberg W., Hightower J., Schiele B., Strang T. Deployment, calibration, and measurement factors for position errors in 802.11-based indoor positioning systems. Proceedings of the Third International Symposium Location and Context-Awareness (LoCA 2007).

[50] Ficco M., Esposito C., Napolitano A. (2014). Calibrating Indoor Positioning Systems with Low Efforts. IEEE Trans. Mob. Comput..

[51] Subedi S., Pyun J. (2017). Practical Fingerprinting Localization for Indoor Positioning System by Using Beacons. J. Sensors.

[52] Zuo Z., Liu L., Zhang L., Fang Y. (2018). Indoor Positioning Based on Bluetooth Low-Energy Beacons Adopting Graph Optimization. Sensors.

[53] Sadowski S., Spachos P. (2018). RSSI-Based Indoor Localization With the Internet of Things. IEEE Access.

[54] Karvonen H., Pomalaza-Ráez C., Mikhaylov K., Hämäläinen M., Iinatt J. (2019). Experimental Performance Evaluation of BLE 4 Versus BLE 5 in Indoors and Outdoors Scenarios. Advances in Body Area Networks I.

[55] Pancham J., Millham R., Fong S., Gervasi O., Murgante B., Misra S., Stankova E., Torre C., Rocha A., Taniar D. Investigation of Obstructions and Range Limit on Bluetooth Low Energy RSSI for the Healthcare Environment. Proceedings of the 18th International Conference on Computational Science and Its Applications (ICCSA 2018).

[56] Peng Y., Niu X., Tang J., Mao D., Qian C. (2018). Fast Signals of Opportunity Fingerprint Database Maintenance with Autonomous Unmanned Ground Vehicle for Indoor Positioning. Sensors.

[57] Nastac D., Lohan E., Iftimie F., Arsene O., Cramariuc B. Automatic Data Acquisition with Robots for Indoor Fingerprinting. Proceedings of the 12th International Conference on Communications (Comm’18).

[58] De Blasio G., Quesada-Arencibia A., Rodríguez-Rodríguez J.C., García C.R., Moreno-Díaz R. Impact of Beacon-Dependent Parameters on Bluetooth Low Energy Indoor Positioning Accuracy. Proceedings of the 12th Conference on Ubiquitous Computing and Ambient Intelligence (UCAmI 2018).

[59] Bulusu N., Heidemann J., Estrin D., Ieee Computer S. Adaptive beacon placement. Proceedings of the 21st IEEE International Conference on Distributed Computing Systems (ICDCS 2001).

[60] Chawathe S. Beacon Placement for Indoor Localization using Bluetooth. Proceedings of the 11th International IEEE Conference on Intelligent Transportation Systems (ITSC 2008).

[61] Ji M., Kim J., Jeon J., Cho Y. Analysis of Positioning Accuracy corresponding to the number of BLE beacons in Indoor Positioning System. Proceedings of the 17th International Conference on Advanced Communication Technology (ICACT 2015).

[62] Kriz P., Maly F., Kozel T. (2016). Improving Indoor Localization Using Bluetooth Low Energy Beacons. Mob. Inf. Syst..

[63] He W., Ho P., Tapolcai J. (2017). Beacon Deployment for Unambiguous Positioning. IEEE Internet Things J..

[64] Castillo-Cara M., Lovon-Melgarejo J., Bravo-Rocca G., Orozco-Barbosa L., Garcia-Varea I. (2017). An Empirical Study of the Transmission Power Setting for Bluetooth-Based Indoor Localization Mechanisms. Sensors.

[65] LEGO Mindstorms. https://www.lego.com/en-us/mindstorms.

[66] ev3dev Home. https://www.ev3dev.org/.

[67] Cha S.H. (2007). Comprehensive Survey on Distance Similarity Measures. Int. J. Math. Models Methods Appl. Sci..

[68] Kontak.io Beacon Configuration Strategy Guide - Transmission Power-Blog. https://kontakt.io/blog/ibeacon-configuration-guide-tranmission-power/.

[69] De Blasio G., Quesada-Arencibia A., Garcia C., Rodriguez-Rodriguez J., Moreno-Diaz R. (2018). A Protocol-Channel-Based Indoor Positioning Performance Study for Bluetooth Low Energy. IEEE Access.

